# Protocol optimization and reducing dropout in online research

**DOI:** 10.3389/fnhum.2023.1251174

**Published:** 2023-12-05

**Authors:** Halee Staggs, Colleen Mills-Finnerty

**Affiliations:** ^1^Mental Illness Research Education and Clinical Center, VA Palo Alto Health Care System, Palo Alto, CA, United States; ^2^Department of Psychiatry and Behavioral Sciences, Stanford University, Palo Alto, CA, United States; ^3^Shiley-Marcos School of Engineering, University of San Diego, San Diego, CA, United States

**Keywords:** online research, research methods, classification model, decision tree, nicotine, cannabis, attrition, diversity

## Abstract

Online research has advantages over in-person research; it’s cost-efficient, scalable, and may increase diversity. Researchers collecting data online can assess protocol performance with classification models like a decision tree. However, challenges include attrition, lack of testing environment controls, technical limitations, and lack of face-to-face rapport and real time feedback. It is necessary to consider human factors of the teleresearch process from recruitment to data collection. Here we document the impact of protocol optimizations on social media engagement and retention between a pilot sample of Veterans (*n* = 26) and a post-optimization sample of both Veterans and civilians (*n* = 220) recruited from Facebook advertisements. Two-sided tests for equality of proportions were statistically significant: advertisement views leading to clicks increased by 23.8% [*X*^2^(1) = 130.3, *p* < 0.001] and completion of behavioral tasks increased by 31.2% [*X*^2^(1) = 20.74, *p* < 0.001]. However, a proportion of participants dropped out of the study before completion for both samples. To explore why, a C5.0 decision tree was used to find features that classify participant dropout. The features chosen by the algorithm were nicotine use (100%) and cannabis use (25.6%). However, for those completing the study, data quality of cognitive performance was similar for users and nonusers. Rather than determining eligibility, participants who endorse using nicotine, or both nicotine and cannabis, may have individual differences that require support in online protocols to reduce drop out, such as extra breaks. An introduction page that humanizes participants’ lifestyle habits as a naturalistic benefit of remote research may also be helpful. Strategies are discussed to increase engagement and improve data quality. The findings have implications for the feasibility of conducting remote research, an increasingly popular approach that has distinct challenges compared to in-person studies.

## 1. Introduction

There has been a proliferation of remote data collection methods that participants can complete from home on their personal computers and devices. Teleresearch is a term used to describe research protocols that incorporate, either fully or partially, digitalized, remote adaptations of traditional, in-person research activities. In-person methods may include flyers or tabling for recruitment, hard copy consents, paper-and-pencil surveys or cognitive assessments, and sending compensation checks in the mail. Teleresearch uses modalities like video interfacing, digital document signing, social media advertising, browser-based data collection, and electronic gift cards. Teleresearch is cost efficient, reaches a wide audience, reduces paper waste, and may reduce barriers associated with in-person data collection like geographic location. Teleresearch methods have been applied in both empirical research (Mills-Finnerty et al., 2021, 2023) and clinical trials (Ahmadi et al., 2018; Badran et al., 2022; Beg et al., 2022; Davies et al., 2022; Simmons et al., 2022). Many aspects of research can be implemented in a remote format, but this comes with challenges, such as high attrition rates of participants who enroll (as much as 25%).

The wider adoption of remote modalities due to COVID-19 has demonstrated that research can be automated and scaled, including recruitment, consenting, data collection, and participant payment. Researchers can reach a nationwide (or worldwide) audience with a cost-effective, social media ad campaign that funnels participants into the data collection process. Teleresearch reduces travel costs, the amount of time dedicated to data collection, and staff salaries, with potential to collect data from hundreds or thousands of people simultaneously. More important than automation, teleresearch may be more generalizable than in-person studies. Compared to convenience samples of college undergraduates or local community members, remote research is not limited by geographic location or socioeconomic barriers. This may increase diversity and subsequent generalizability of research findings because datasets may be less homogenous. Teleresearch reaches people who live in rural areas (14% of our sample), people with mental health conditions (38% of our sample), and special populations such as Veterans (24% of our sample). However, people without Wi-Fi (9.7% of US population; United States Census Bureau, 2021), those with unreliable electricity, and people who lack computer literacy skills will not be easy to recruit for teleresearch studies. However, information can be collected from most populations with remote methods, and this improves the usefulness of scientific efforts. Peer-reviewed, empirical assessments of the impact of teleresearch on diversity are needed.

Despite the many benefits of teleresearch, several limitations exist. First, internet-based research may be vulnerable to bot algorithms that complete studies with web-scraped data. It is important to incorporate and validate tests of human capacity throughout data collection (language proficiency, creativity, reCAPTCHA). Identity for groups, like Veterans, can also be verified in medical records if the study consent includes such a query. However, with the recent proliferation of natural language processing algorithms, such as ChatGPT (OpenAI, 2023), online researchers are encouraged to verify human identity via a phone call or Zoom interaction, especially when identity cannot be verified through other methods. This study was completed prior to the release of ChatGPT. Another potential limitation is lack of environmental control. Unlike a lab environment where a researcher can provide feedback, it is impossible to know if online participants are inattentive or experiencing frustration. However, these concerns can be mitigated with intentional research design. The teleresearch study becomes an object that participants are interacting with, and that object must be interesting and worthwhile to get complete, high-quality data. With an engaging protocol, the naturalistic testing environment can become a benefit of teleresearch by reducing performance anxiety, demand characteristics, or personal discomfort or embarrassment while providing sensitive information in the presence of research staff, such as details about sexual performance, trauma, or substance use. Participants might be more forthcoming with such questions in the privacy of the home. A third limitation of online research is participant attrition. Participants drop out at different points throughout the data collection process, possibly because closing their browser and ending participation is easier than leaving an in-person session. It is necessary to characterize drop out by finding design weaknesses or participant features with data-driven methods. A final limitation to consider with online research is institutional regulations for privacy and data security (e.g., HIPAA). Protected health information can only be collected and stored in secure environments. Digital data privacy can introduce complexities into the data collection methods.

The primary purpose of this study was to assess cognition in Veterans and civilians both with and without depression. The teleresearch aspects of this protocol included social media advertising on Facebook, a website sign-up page with a prescreening survey, DocuSign for consent, and a browser-based data collection workflow for self-report measures and cognitive tasks. A recruitment pilot sampled was collected to establish feasibility which revealed slow recruitment, user complaints, and incomplete datasets. Following the pilot sample, several updates were made to optimize the protocol and to build-in a data quality support system. For recruitment, the aim of optimization was to increase social media advertisement engagement and reduce the number of ineligible participant sign-ups. For enrollment, the aim of optimization was to increase retention between consenting and subsequent data collection. In this secondary analysis, we report the impact of such optimization strategies on retention rates between the two teleresearch protocols. Because this is a retroactive, secondary analysis, we cannot control for the updates as separate variables. We quantify the effects of the protocol optimization using two-sided, two-sample tests for equality of proportions between the pilot sample and the post-optimization sample. Despite improvements in retention, drop out still occurred in the post-optimization sample, but at a lower rate for key measures.

We aimed to uncover what demographic and substance use features (nicotine, cannabis, alcohol) classify participant drop out using a decision tree (Kuhn and Johnson, 2013). The surveys capturing these variables were administered first, and thus had nearly perfect completion rates. Teleresearchers can use descriptive machine learning (ML) algorithms, like decision trees, to uncover insights and key metrics within a dataset, also known as data mining. Classification decision trees are groups of if-then statements that separate data into groups that classify an outcome (Kuhn and Quinlan, 2023). One type of decision tree is the C5.0 algorithm which can handle multi-categorical and numerical data as predictor variables (Quinlan, 1993; Quinlan, 1996; Kuhn and Quinlan, 2023) and has been used across many industries to classify groups (Ahmadi et al., 2018; Elsayad et al., 2020; Tian and Zhang, 2022; Wati et al., 2022; Delgado-Gallegos et al., 2023). All variables from a dataset can be given to the algorithm and it will choose the most important features that classify the outcome and discard the irrelevant features (Kuhn and Quinlan, 2023). The C5.0 algorithm is computationally robust, and the results are easy to interpret both graphically and logically, making it a good choice to explore relationships within datasets. This approach considers the whole sample and uses the entire dataspace, which can be more insightful than traditional statistical testing where feature distributions are collapsed into means. A novel use case for C5.0, the algorithm was used here to decide what variable values separate study completers from those who dropped out (i.e., binary classification). The nodes of the tree contain characteristics of participants that may need extra support during participation to prevent drop out. The findings have implications for the feasibility of conducting remote research, an increasingly popular approach that has distinct challenges compared to in-person studies.

## 2. Method

### 2.1. Sample

Participants (*N* = 246; *n* = 26 pilot; *n* = 220 post-opt) were recruited with Facebook advertising with pop-art graphics containing Stanford University and US Department of Veterans Affairs logos. The study recruited Veterans and civilians (see Funding) both with and without depression. The pilot sample was collected in March and April of 2022 during a 6-week ad campaign, and the post-optimization sample was collected from June 2022 to October 2022 with a total of 9 weeks of ads. The advertisements contained a link to our study website page where participants completed a brief screening survey (see Workflow sections below for screening questions). If participants pass the screening, they are directed to the sign-up page and report their contact information. All participants were over 18 years old, completed informed consent in DocuSign, and passed an English fluency assessment task. There were no targets for demographics, however, the sample mirrors race and education proportions of the general US population (Table 1; United States Census Bureau, 2021). In contrast, the sample contains a higher prevalence of nicotine (Center for Disease Control and Prevention, 2023) and cannabis users (Center for Disease Control and Prevention, 2021), and people with disabilities compared to census data (United States Census Bureau, 2021). Veterans and civilians were found to differ with respect to sex and age (Table 2) but were similar for all other demographics. The differences in study protocols are described below and listed in Table 2. The study protocol was approved by the Stanford Institutional Review Board (protocol 47906).

**Table 1:** Sample demographics compared to US census data (United States Census Bureau, 2021) and CDC data (Center for Disease Control and Prevention, 2021, 2023).

Demographic	Level	PilotV n/p̂	PostV n/p̂	PostC n/p̂	All n/p̂	Census p̂	CDC p̂
Age	−	56.9 ± 9.9	51.9 ± 12.3	44.1 ± 14.7	46.5 ± 14.5	−	−
Type	Veteran	26/100.0	34/100.0	0/0.0	60/24.4	6.4	*
	Civilian	0/0.0	0/0.0	186/100.0	186/75.6	93.6	*
Sex	Male	21/80.8	22/64.7	63/33.9	106/43.1	49.5	*
	Female	5/19.2	12/35.3	123/66.1	140/56.9	50.5	*
Ethnicity	Non-Hisp	24/92.3	32/94.1	167/89.8	223/90.7	81.1	*
	Hispanic	2/7.7	2/5.9	19/10.2	23/9.3	18.9	*
Area type	Urban	10/38.5	12/35.3	67/36.0	89/36.2	*	*
	Suburban	15/57.7	19/55.9	88/47.3	122/49.6	*	*
	Rural	1/3.8	3/8.8	31/16.7	35/14.2	*	*
Psych Hist	No	14/53.8	25/73.5	114/61.3	153/62.2	87.0^a^	*
	Yes	12/46.2	9/26.5	72/38.7	93/37.8	13.0^a^	*
Race	White	18/69.2	22/64.7	143/76.9	183/74.4	75.8	*
	Black	4/15.4	9/26.5	20/10.8	33/13.4	13.6	*
	Asian	1/3.8	2/5.9	14/7.5	17/6.9	6.1	*
	Multiple	2/7.7	1/2.9	6/3.2	9/3.7	2.9	*
	Native^b^	0/0.0	0/0.0	3/1.6	3/1.2	1.3	*
	Unknown	1/3.8	0/0.0	0/0.0	1/0.4	*	*
Degree	HS/GED	6/23.1	3/8.8	43/23.1	52/21.1	27.9	*
	AA/S	6/23.1	10/29.4	28/15.1	44/17.9	10.5	*
	BA/S	8/30.8	13/38.2	58/31.2	79/32.1	23.5	*
	MA/S	3/11.5	7/20.6	45/24.2	55/22.4	14.4^c^	*
	PhD/MD	3/11.5	1/2.9	11/5.9	15/6.1	14.4^c^	*
	None	0/0.0	0/0.0	1/0.5	1/0.4	8.9	*
Nico use	No	18/69.2	24/70.6	141/75.8	183/74.4	*	88.5
	Yes	8/30.8	10/29.4	45/24.8	63/25.6	*	11.5
Cann use	No	17/65.4	24/70.6	127/68.3	168/68.3	*	82.0
	Yes	9/34.6	10/29.4	59/31.7	78/31.7	*	18.0
Dropout	No	10/38.5	29/85.3	153/82.3	192/78.0	*	*
	Yes	16/61.5	5/14.7	33/17.7	54/22.0	*	*

*Is an unavailable census or CDC value; nico, nicotine; cann, cannabis; PilotV, pilot sample of all Veterans; PostV, post-optimization Veterans; PostC, post-optimization civilians.

a The census data reports that 13% of the US population has some form of disability: whether mental or physical. Psychiatric history reported here.

b Alaskan native or native American. The study sample does not include native Pacific Islanders.

c Percentage of adults with a Master’s degree or higher.

**Table 2:** Statistical tests for differences between Veterans and civilians.

Demographic	*t*	*X* ^2^	*df*	*p*
Age^*^	3.70	−	67.5	<0.001
Sex^*^	−	13.10	1	<0.001
Race	−	4.77	4	0.31
Ethnicity	−	0.07	1	0.78
Area	−	1.60	2	0.45
Psych Hist	−	0.44	1	0.51
Degree	−	7.65	5	0.18
Nico use	−	0.02	1	0.88
Cann use	−	0.54	1	0.46

* Statistically significant difference.

### 2.2. Workflow for the pilot sample

Pilot participants were shown Facebook advertisements with headlines including the word “Veteran”. The sign-up website advertised compensation for $5.00. The compensation amount was determined based on crowdsource platforms (MTurk, Prolific) recommendations for study payments during the period of data collection. The following inclusion criteria were assessed in the screener: Veteran or civilian (only Veteran allowed through due to targeting special population), and age 18 or older. If they pass the screening, they can add their contact information. Participants consented in DocuSign via an email invitation. REDCap (Harris et al., 2009, 2019) links were emailed which automatically linked to the subsequent two data collection platforms (Table 3).

**Table 3:** Protocol workflows for both samples.

Pilot	Post-optimization
*Recruitment*	*Recruitment*
Facebook Ad: Veterans	Facebook Ad: Veterans and civilians^*^
Website: $5.00 compensation	Website: $15.00 compensation^*^
Pre-Screen: 2 Questions	Pre-Screen: 8 Questions^*^
Sign-Up page	Sign-UP page
DocuSign request	Introductory email^*^
Email study link	DocuSign request
	Email study link with reminders^*^
*Data collection*	*Data collection*
*Part 1: Surveys in REDCap*	*Part 1: Surveys in REDCap*
Veteran verification	Study team intro and study map^*^
Demographics	Veteran verification
Health History	Demographics
PROMIS PF-12	Health history
BIS/BAS	PROMIS PF-12
MASQ-62	BIS/BAS
DEBQ	MASQ-26^*^
PHQ-8	DEBQ
GAD-7	
*Part 2: Gorilla tasks*	*Part 2: Gorilla tasks*
Intro questionnaire	Intro questionnaire
Delayed discounting	General instructions^*^
Flanker	T/f attention check questions^*^
Willingness to pay	1 minute break^*^
Visual search	GAD-7
Simon’s	Travel preferences^*^
	Delayed discounting
	PHQ-8
	Visual search^*^
	Willingness to pay
	1 minute break^*^
	Flanker^*^
Part 3: Unity task	Part 3: Unity task

* Denotes a task that was optimized or added, see text descriptions.

### 2.3. Workflow for the post-optimization sample

Post-optimization participants were shown Facebook advertisements with neutral headlines to target both Veterans and civilians. The study was described as a decision making study. The sign-up website advertised compensation up to $15.00, with the surveys being $5.00 and the cognitive tests for an additional $10.00. The following inclusion criteria were assessed in the screener: Veteran or civilian (both allowed through – target population and comparison group), age 18 or older, reliable Wi-Fi, access to a computer with a keyboard, distraction-free environment to complete the study, up-to-date computer operating system, confirmation of understanding that DocuSign will be used for informed consent (with a note that instructions will be sent via email), and confirmation of understanding that a link to start the study will be emailed after providing consent. If they pass the screening, they can add their contact information. Participants were emailed two emails: general instructions for study tasks and a DocuSign invitation. REDCap (Harris et al., 2009, 2019) links were emailed which automatically linked to the subsequent two data collection platforms (Table 3).

### 2.4. Self-report data in REDCap

In part one of the study, participants completed a series of self-report surveys in REDCap (Harris et al., 2009, 2019). The primary outcome of the study was to assess how self-reported mental health symptoms relate to cognitive performance. To begin, participants are asked to report their Veteran status. For those reporting to be a Veteran, they must pass follow-up questions to be allowed to continue in the study (i.e., insider knowledge). Following that, participants complete surveys for demographics, health history, and medications. For the pilot sample, we used the PROMIS Physical Function-12 (Fries et al., 2011), Behavioral Inhibition and Activation Scales (Carver and White, 1994), Mood and Anxiety Symptom Questionnaire-62 (Watson et al., 1995), Dutch Eating Behavior Questionnaire (Van Strien et al., 1986), Patient Health Questionnaire-8 (Kroenke et al., 2009), and Generalized Anxiety Disorder-7 (Spitzer et al., 2006). For the post-optimization sample, we used the 26-item version of the MASQ (Watson et al., 1995), and the PHQ-8 (Kroenke et al., 2009) and GAD-7 (Spitzer et al., 2006) were administered in the second platform. All other measures were the same and were automatically scored with calculation fields in REDCap (Supplement 1).

### 2.5. Cognitive testing in Gorilla Experiment Builder

Following the completion of REDCap (Harris et al., 2009, 2019), participants were automatically linked to part two: a customized cognitive testing battery in Gorilla Experiment Builder (Anwyl-Irvine et al., 2020, 2021). The cognitive battery data is for the primary analysis aims of the study. A computer with a keyboard was required for the cognitive tasks due to specific button key responses. Participants were informed of this requirement in the pre-screen survey, the consent form, the introduction email, the introduction page of the Part 1, and the screen prior to the link transfer to the cognitive tasks, whereon participants were told to switch devices now if they were not using a computer with a keyboard. Non-compatible device use was identified because participants would time-out on the task requiring button key presses. The battery assessed the following cognitive domains: reaction time, impulsivity, visual attention, reward sensitivity, and accuracy. The tasks included custom adaptations of Visual Search (Eriksen, 1995): blue and orange letter arrays with target symbol being absent or present, Flanker (Eriksen and Eriksen, 1974): a target cartoon fish facing left or right with flanking fish being congruent or incongruent, Willingness to Pay (Plassmann et al., 2007): bidding on food items, Simon’s stimulus–response compatibility (Cespón et al., 2020): the words “Right” and “Left” appearing on alternating sides of the screen being congruent or incongruent, and Delayed Discounting (da Matta et al., 2012; Xia et al., 2017): preference for immediate or delayed monetary rewards.

The pilot sample completed an introductory questionnaire where they input their study ID, 27 trials of Delayed Discounting, 48 trials of Flanker, 56 Trials of Willingness to Pay, 24 trials of Visual Search, and 36 trials of Simon’s (Table 3). The battery contained brief instructions and “start” and “next” buttons to move through tasks.

The post-optimization sample completed an introductory questionnaire where they input their study ID and then read some general instructions for the cognitive tasks. The next screen contained two True/False attention check questions to confirm that they read the instructions on the previous screen. Then a one-minute mandatory break was given. Following the first break, participants completed the GAD-7 (Spitzer et al., 2006), a filler task about travel preferences, 27 trials of Delayed Discounting (da Matta et al., 2012), the PHQ-8 (Kroenke et al., 2009), three practice trials of Visual Search then 24 real trials, and 56 trials of Willingness to Pay (Plassmann et al., 2007). Then a one-minute mandatory break was given again. After the second break, participants completed four practice trials of Flanker then 48 real trials (Eriksen and Eriksen, 1974; Table 3). The prompts for all tasks were updated to have conversational language, and debrief feedback was given at the completion of each task. The practice trials also contained feedback, so participants knew whether they answered correctly or not. The additional details were implemented to increase engagement due to losing participants during the cognitive tasks in the pilot (Supplement 2). The Simon’s task (Cespón et al., 2020) was removed due to cognitive domain redundancy from the other tasks.

### 2.6. Reward sensitivity task in Unity

Participants were automatically linked to the third platform after completing the Gorilla battery (Anwyl-Irvine et al., 2020, 2021): a browser-deployed task coded in Unity. This task data is not required to complete the primary aims of the study but will be used for exploratory purposes in the subgroup that completed it. Participants were required to rate food stimuli on a scale of strongly dislike to strongly like and were subsequently asked to find a target object amongst arrays of highly rated food items (3–8 distractors). This task assessed value-driven attentional priority (Anderson, 2017). The cohorts completed the same version of this task (Supplement 3) whereby responses corresponded to target’s position in the grid array using keyboard numbers 1, 2, 3, 4, 7, 8, 9, and 0.

### 2.7. Recruitment optimization updates and intended purposes

Following the pilot, the following issues were identified: low social media engagement and a high rate of dropouts due to participant hardware incompatibilities. To address this, recruitment was opened to both our target population (Veterans) and the comparison group (civilians) at the same time. The social media advertisement headlines were updated with the word “Veteran” removed, and the target audience settings in Facebook were expanded to include civilian and Veteran users. Compensation was increased from $5.00 to $15.00. The post-optimization sample was offered $5.00 for the surveys, and an additional $10.00 for completing the cognitive tasks, to incentivize completion. Second, the screening survey was expanded to include confirmation of understanding of technical set-up expectations: reliable Wi-Fi and a computer with a keyboard. Many participants from the pilot sample attempted to complete the study on a smartphone, so their data could not be used. Adding these expectations allowed recruitment of eligible participants only. If a participant from the post-optimization sample still attempted to complete the study on a smart phone, they would not be able to finish the cognitive tasks. These participants were contacted to redo the study on a proper device. Participants lost to follow up due to technical incompatibilities were not included in analyses.

### 2.8. Retention optimization updates and intended purposes

For retention, protocol adjustments increased personalization and engagement. The first page of the study was an introduction page to meet the research team. This emulates meeting in person and builds rapport by showing that real people are running the online study. Second, a study map was shown to set expectations and highlight that breaks will be given. People can plan their “distractions” to minimize multitasking. Third, the MASQ (Watson et al., 1995) was updated to its shortest version. Fourth, two 1-min breaks were added to the cognitive testing battery because it is repetitive and challenging. Fifth, the two mental health surveys were added in-between cognitive tasks. The change in task order was meant to reduce repetition of task formats to help participants maintain attention. Sixth, practice trials were added to the accuracy-based cognitive tasks so participants understood what to do. Seventh, entertaining filler questions were added (e.g., questions about travel preferences). Eighth, two truth/false engagement questions were added that asked about instructions to make sure participants were being attentive. Ninth, conversational language was used in task instructions. Tenth, all tasks had encouraging feedback at the end of the task, to make study feel more interactive.

### 2.9. Data quality system

For the post-optimization sample, a system was implemented to assign a score to participants’ data quality based on effort and attention. A composite quality score was composed of seven possible points for people who completed the entire study. This assessment will be used to guide the data cleaning process for this study’s primary analysis plan.

1) One point was given for a topic-related response to the following question: In one to two sentences, tell us your favorite animal and why?
2) Participants were shown their ID number at the end of part one of the study and were instructed to input the number of the first screen of part two of the study. Participants were given one point for following this instruction properly.
3) One point was given for answering the first True/False question about study instructions correctly.
4) One point was given for answering the second True/False question about study instructions correctly.
5) One point was given for a topic-related response to the following question: In one to two sentences, tell us your favorite vacation photo and why? Participants were shown three images.
6) One point was given for having a reaction time standard deviation within 1.5 times the interquartile range for the sample for the visual search task. Participants with extremely low standard deviations were assumed to be bots (extreme response consistency), and participants with extremely high standard deviations were assumed to lack effort (extreme response inconsistency).
7) One point was given for having a reaction time standard deviation within 1.5 times the interquartile range for the sample for the flanker task. Same assumptions as number six.

## 3. Results

All data analysis was completed in R Studio version 4.2.3 (R Core Team, n.d.) on a PC running Windows 11 using the following packages: tidyverse 1.3.2 (Wickham et al., 2019), ggplot2 3.4.0 (Wickham, 2016), C50 0.1.8 (Kuhn and Quinlan, 2023), stats 4.2.3 (R Core Team, n.d.), caret 6.0.93 (Kuhn, 2022), pwr 1.3.0 (Champely, 2020). Supplement 4 contains the highlights of the code. See the following GitHub repository for all code: https://github.com/HNStaggs/Participant_Dropout_Classification. The following results show the statistically significant improvements between the pilot sample (*n* = 26) and the post-optimization sample (*n* = 220) for recruitment and retention. A C5.0 decision tree shows important participant characteristics related to dropping out for the whole sample. Participants who dropped out because of technical incompatibilities were not included in any analyses. Power calculations (0.8, *p* = 0.05) were ran for all tests and the sample sizes justify moderate to strong effect sizes for this exploratory analysis.

### 3.1. Proportion testing

Two-sided, two-sample tests for equality of proportions were conducted to examine the difference in retention between the pilot sample and the post-optimization sample. The proportion tests are divided into the recruitment period and the enrollment period. There was potential for dropout at multiple points, so retention was examined at each step in the study timeline (Table 4). The recruitment period tests total advertisement views leading clicks to our website, clicks leading to screens, screens leading to sign-ups, and sign-ups leading to consents. The enrollment period assesses retention rates: consents leading to part one, part one leading to part two, and part two leading part three (Table 4).

**Table 4:** Frequency table showing proportion of retention between study components.

Sample		Recruitment			Enrollment			Conversion
		Clicks/Ads	SignUp/Clicks	Consent/SignUp	Part1/Consent	Part1/Part2	Part2/Part3	Part3/Ads
Pilot	*n*	167/628	109/167	36/109	26/36	16/26	10/16	10/628
	*p̂*	26.6*	65.3*	33.0*	72.2	61.5*	62.5	1.6
Post-Opt	*n*	3740/7422	1476/3740	282/1476	220/282	204/220	127/204	127/7422
	*p̂*	50.4*	39.5*	19.1*	78	92.7*	62.3	1.7

* Statistically significant difference.

#### 3.1.1. Recruitment period

The amount of clicks in proportion to total ad views increased by 23.8% after updating the compensation amount and opening recruitment to both groups [pilot 26.6%; post-opt 50.4%; *X*^2^(1) = 130.3, *p* < 0.001]. The increased activity with our ads had the intended affect: boosting activity in the Facebook algorithm. The amount of sign-ups in proportion to clicks decreased by 25.8% [pilot 65.3%; post-opt 39.5%; *X*^2^(1) = 43.1, *p* < 0.001] and the amount of consents in proportion to sign-ups decreased by 13.9% (pilot 33.0%; post-opt 19.1%; *p* < 0.001). The updated screening survey and the information on the website had the intended effect: to deter more ineligible people from signing up compared to the pilot sample.

#### 3.1.2. Enrollment period

There was a nonsignificant difference in the proportion of retention between consenting and part one for both samples (pilot 72.2%; post-opt 78.0%; *p* = 0.568). A similar number of participants attempted to start the study after consenting by clicking on the REDCap link sent via email. The main outcome measure was to correlate mental health symptoms with cognitive performance. As such, it was imperative that participants completed part one (REDCap) and part two (Gorilla Experiment battery). The amount of participants completing part two in proportion to part one increased by 31.2% after optimizing the protocol for engagement [pilot 61.5%; post-opt 92.7%; *X*^2^(1) = 20.7, *p* < 0.001]. Participants were equally likely [*X*^2^(1) < 0.001, *p* = 1] to drop out between part two and three for both samples (pilot 62.5%, post-opt 62.3%). Because part 3 was an exploratory aim, this did not affect the primary aim analysis plan. However, the consequence of using too many platforms could be higher chance of technical difficulties or boredom.

### 3.2. Classification model for participant drop out

**Figure 1:**
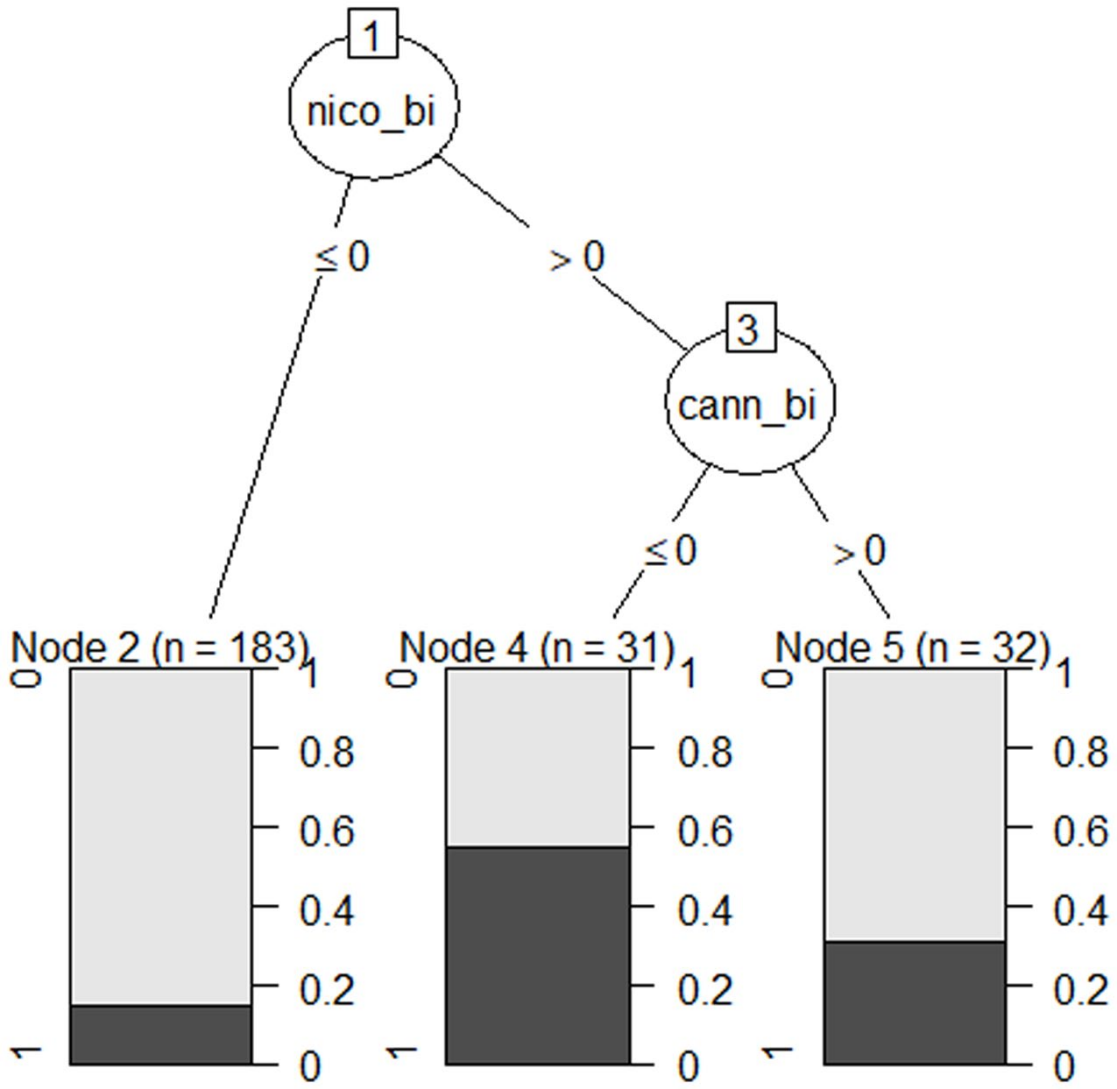
C5.0 decision tree model plot for participant drop out.

A C5.0 decision tree model (Kuhn and Quinlan, 2023) was used to classify participant dropout (0 = Completer; 1 = Dropout) between platform 1 and platform 2 (Figure 1) for the full sample (*N* = 246). Participants were instructed to complete the study in one sitting; however, they had unlimited time for the surveys and 24 h for the cognitive tasks. This allowed some flexibility in case they stepped away from their browsers. If a participant stopped participation prior to completion, they were contacted with an offer to continue where they left off. Participants labeled as dropouts did not respond to follow up attempts. Due to the explanatory use of the C5.0 decision tree, the small sample size (Kuhn and Johnson, 2013), and the small number of predictors, the model could not be boosted, and performance was not validated with a test set. The input variables given to the algorithm were age, sex, ethnicity, race, residence area type, veteran status, education, nicotine use, cannabis use, alcohol use, and psychiatric history. With a minimum of 8 participants in each node to maintain statistical power, the primary split chosen by the algorithm was nicotine use (100% importance; No ≤0; Yes >0) with one additional split for cannabis use (26.3% importance; No ≤0; Yes >0). All other demographic factors were discarded by the algorithm meaning they were irrelevant to the classification of dropout. Twenty-seven people out of 183 (14.8%) who do not use nicotine dropped out of the study (node 2), 17 people out of 31 (54.8%) who use only nicotine but not cannabis dropped out of the study (node 4), and 10 people out of 32 (31.3%) who use both substances dropped out of the study (node 5). The proportion of drop out is denoted by the darker color in the node description. It is important to note that not all people who use cannabis and/or nicotine dropped out of the study, yet the number who did represent a significant proportion of the subgroup. When looking at the subgroup dropout proportions, the highest rate is for people who endorse using nicotine only, with a slightly lower rate for both nicotine and cannabis use, with the lowest rate being those reporting no nicotine use. Conclusions cannot be made for people who endorse only cannabis use from this model.

#### 3.2.1. Data quality analysis

For those that completed the study (*n* = 190), one-way analyses of variance (group x reaction time) were conducted to test whether people who endorse nicotine or cannabis use (*n* = 74) have different reaction time distributions compared to nonusers (*n* = 116). Reaction times were log-transformed. The results were non-significant for the Visual Search task’s absent [*F*(1, 120) = 0.18, *p* = 0.67] and present [*F*(1, 126) = 0.063, *p* = 0.80] conditions and the Flanker task’s incongruent [*F*(1, 163) = 0.031, *p* = 0.86] and congruent [*F*(1, 163) = 0.017, *p* = 0.90] conditions. The results of the Kruskal tests (group × accuracy) were also non-significant for Visual Search conditions [absent, *X*^2^(1) = 0.95, *p* = 0.33; present, *X*^2^(1) = 2.69, *p* = 0.10] and Flanker conditions [incongruent, *X*^2^(1) = 1.01, *p* = 0.32; congruent, *X*^2^(1) = 0.29, *p* = 0.59]. There was a non-significant difference in data quality scores (*n* = 179 available) between the user and nonuser group [*F*(1, 177) = 0.23, *p* = 0.63]. Because data quality was similar between users and nonusers, nicotine and cannabis use may be solely related to chance of drop out, and not participant effort or eligibility. Due to the exploratory nature of this analysis, additional variables were not controlled for, such as age.

## 4. Discussion

Teleresearch is an accessible data collection format that has many benefits for researchers and participants. Despite the advantages, teleresearch has limitations that require creative solutions to maximize retention and assess data quality. This paper detailed the impact of protocol optimizations following a pilot testing phase. The following aspects of pilot protocol were suboptimal: social media engagement, technical set-up compatibilities, and retention between platforms. Many aspects of the study design were optimized to reduce drop out and increase usable data. The overall conversion from advertisement views to platform three was similar for both samples (pilot 1.6%, post-opt 1.7%), however, a combination of three key metrics resulted in more usable data for the study’s primary aims in the post-optimization sample. The updated protocol had increased social media engagement (23.8%) followed by more thorough screening (19.1% of sign-ups were eligible compared to 33% in the pilot), leading to a significant increase in the amount of usable data collected (31.2% increase in completion of Part 2). Data-driven evaluation of protocol performance allows researchers to identify weaknesses in remote study design. Variables can be identified that classify drop out at each protocol step using machine learning methods like decision trees. Quantifying protocol efficiency should be part of preregistered data analysis plans.

Despite significant improvements in the protocol, some drop out still occurred post-optimization. Demographic factors associated with dropping out were identified using a C5.0 classification decision tree. The algorithm (Kuhn and Johnson, 2013; Kuhn and Quinlan, 2023) was used because it provides an easily interpretable decision tree that ignores irrelevant variables from the data. Because the results of decision trees will change based on small changes in datasets or imbalanced classes, we used it as an exploratory tool paired with domain expertise, to reveal factors that impacted retention. The model identified that a greater proportion of people who use only nicotine dropped out of the study compared to people who use both nicotine and cannabis, and both groups had higher dropout percentages compared to nonusers of nicotine. The interpretation for cannabis-only users cannot be determined from the model. People who use these substances may have difficulties with impulsivity (Round et al., 2020; Spechler et al., 2020; Yan et al., 2021), attention, processing speed (Vangkilde et al., 2011), or working memory (Lisdahl et al., 2016). People who work online for money may also have more mental health challenges compared to people who volunteer to come in for studies in-person (Mills-Finnerty et al., 2021, 2023), considering rates of depression and anxiety were higher than census values in our sample (United States Census Bureau, 2021). People with depression, anxiety, or ADHD may also have a higher chance of substance use (Xu et al., 2021). Researchers should be developing detailed screening protocols so these variables can be controlled for in analysis plans. We are not suggesting that studies should screen out nicotine and cannabis users, but rather that researchers should be aware of the characteristics of participants who complete studies online and build in support to the protocol. Considerations such as frequency of use and symptoms of dependence may be relevant. Nicotine can have positive effects on attention acutely (Valentine and Sofuoglu, 2018), but factors such as craving and withdrawal may be distracting for particularly heavy smokers. Future studies might ask follow-up questions of those who use tobacco or marijuana to quantify usage patterns to determine how this impacts performance within the study. For example, the average completion time for our study was 45 min, so this amount of time may have been hard to sustain for people with attentional deficits. It may also be helpful to humanize participants’ lifestyle habits on the first screen of the study. Educating participants about the benefits of the naturalistic setting may reduce embarrassment of drop out due to substance use and may increase the chance of participants communicating to continue the study. Notably, there are 27 people who dropped out of the study for reasons that were not captured by the algorithm (i.e., no nicotine use). Some drop out is random, which is likely, but we also lack the appropriate data to model all drop out reasons. The purpose of this analysis was to gain insight into demographics that can be intervened upon, and we identified that it may be harder to retain people who endorse using nicotine only, or who use both nicotine and cannabis, compared to people who do not use nicotine. For the nicotine and cannabis users who completed the study, data quality was comparable to nonusers. Despite potential distractions in the home environment, participants using these substances provide usable data. Thus, use of these substances should not determine study eligibility but can be accommodated in study design choices.

This secondary analysis has several limitations. First, the advertisement period for the pilot group was 3 weeks shorter (6 weeks) than the period for the post-optimization group (9 weeks). However, the amount of advertisement views increased 12-fold after updating the protocol, showing that there was an exponential, not linear, relationship between ad time and views. This is likely due to increased social engagement in the Facebook algorithm, which was a primary goal for the optimization. Second, the pilot group was all Veterans, and the post-optimization group included both Veterans and civilians. There may be an effect of target population that is not accounted for in our analyses. However, Veteran status was used as an input variable in the decision tree, and the algorithm chose to discard the variable as irrelevant to the classification of drop out. This provides evidence that Veteran status may not be a factor for drop out in this sample, which is considered a key insight for the study team. In general, analyses comparing the effect of two different interventions on two groups should ensure that the groups are similar, however, the exploratory use of the decision tree was still insightful for future protocol design choices. Third, implications of the decision tree model are insightful but do not imply causation. Nicotine and cannabis use are likely a proxy variable for other individual differences; it is still uncertain why people dropped out. Additionally, it would have been more useful to use the decision tree as a tool throughout the data collection period. This could have given real time insight compared to post-hoc speculation. Aside from complaints or questions, participant feedback was not solicited. Fourth, the results of the decision tree may not be fully generalizable to the community because of the higher rates of substance use compared to census data. Comparing census data to our sample’s data may also not be ideal because the characteristics of people participating in the census may be different than people interacting with depression research studies advertisements on Facebook. However, data was collected from people both with and without depression, and from both Veterans and civilians, so a significant portion of the sample has variance in the data that resembles the census numbers. With the higher numbers of nicotine and cannabis use, the decision tree model had an appropriate amount of data to find group differences. Studies recruiting non-clinical populations may not be affected by a high number of nicotine or cannabis endorsers. Fifth, there may be other factors that are relevant for drop out that could not be assessed in this analysis. Future analyses should assess more variables like socioeconomic status, family support, or income, and it is recommended that these data be collected first in the protocol to get the highest completion rates. Sixth, remote, digital data collection methods were possible even before the pandemic. However, the adoption of teleresearch methods has been slow within the Department of Veterans Affairs. We acknowledge that these methods may not be as novel to researchers working in the private sector, but that thoughtful teleresearch design and dropout evaluation is a benefit to the field of human research in general. Finally, it is important to acknowledge that it is difficult or impossible to eliminate attrition online particularly for behavioral studies not providing a treatment. Researchers should expect to overrecruit by at least 15–20%.

We show here that classification algorithms can be used as part of the data quality assessment for teleresearch protocols. Future teleresearch studies can use the C5.0 algorithm on demographic variables, self-report data, and numerical metrics (e.g., reaction time) during a pilot phase to identify variables related to attrition rates. While the algorithm itself is robust, it is easy to implement and to interpret, and is a useful exploratory tool for researchers doing work online. Additionally, future teleresearch studies may want to administer a participant feedback form. This form could assess if environmental distractions occurred, if participants thought the study was interesting or fun, if they were confused about anything, if they experienced technical difficulties, what they thought was easy or difficult, or if the study had any mental health triggers. Perspectives from the participants can translate to interesting input variables for a C5.0 model, especially because the motivation of people doing work online may be different than people recruited for in-person studies. There may be socioeconomic or environmental factors related to dropping out that we are unaware of. Studies should attempt to quantify such metrics and should humanize the participants’ experience, including recognition of the potential need for smoke breaks.

This paper detailed many important aspects of designing and evaluating the performance of a teleresearch protocol. Data mining can be used to uncover insights for study design improvements and data quality assessments. Data-driven approaches can diagnose issues at each step in the research process and are especially important for teleresearchers that rely on automated systems that lack human interaction. Here we quantified the success of protocol optimizations for key outcome measures and used a decision tree model in a novel way to classify participant dropout based on demographic input features. Statistical evidence shows that a pilot testing phase is essential for teleresearch studies. Researchers collecting data online should assess protocol performance with data-driven, systematic methods. It is important to document the successes and failures of online research strategies so a shared knowledge base can be developed in this transformational era.

## Data availability statement

The raw data supporting the conclusions of this article will be made available by the authors, without undue reservation.

## Ethics statement

The studies involving humans were approved by Stanford University Institutional Review Board. The studies were conducted in accordance with the local legislation and institutional requirements. The participants provided their written informed consent to participate in this study.

## Author contributions

HS: data collection, data analysis, and manuscript preparation. CM-F: study design and manuscript preparation. All authors contributed to the article and approved the submitted version.
