# Supplementary material for: Protocol optimization and reducing dropout in online research
Source: Front Hum Neurosci. 2023 Dec 5;17:1251174. doi: 10.3389/fnhum.2023.1251174 (PMC10729001; doi:10.3389/fnhum.2023.1251174)
Supplement: Supplementary file 1 [file Table_1.docx]

# Load libraries for data analysis

library(tidyverse)

library(car)

library(C50)

library(caret)

# Proportion Testing

prop.test(x = c(167, 3740),

n = c(628, 7422)) #Ads to clicks

prop.test(x = c(109, 1476),

n = c(167, 3740)) #Clicks to SignUps

prop.test(x = c(36, 282),

n = c(109, 1476)) #SignUps to Consent

prop.test(x = c(36, 282),

n = c(109, 1476)) #Consents to Part 1

prop.test(x = c(26, 220),

n = c(36, 282)) #Part 1 to Part 2

prop.test(x = c(16, 204),

n = c(26, 220)) #Part 2 to Part 3

prop.test(x = c(10, 127),

n = c(628, 7422)) #Ads to Part 3

# Create drop out variable for people who do not have a Gorilla ID (i.e., no cognitive data)

data$gor_id[is.na(data$gor_id)] <- 0 #update missing ID values to a 0

data$dropout <- if_else(data$gor_id == 0, 1, 0) #Assign value of 1 (Yes-Dropout) to 0 Ids

# Update Nicotine and cannabis Use to Binary Variables for Yes/No 0/1

data$nico <- as.numeric(data$nico)

data$cann <- as.numeric(data$cann)

data$nico_bi <- if_else(data$nico > 1,1,0)

data$cann_bi <- if_else(data$cann > 1,1,0)

data$nico_bi <- as.factor(data$nico_bi)

data$cann_bi <- as.factor(data$cann_bi)

# C5.0 Decision Tree

set.seed(2) #set seed for reproducibility

c50 <- C5.0(formula = dropout ~ . , data = data) #Dropout predicted from all variables in random sample

summary(c50) #check out model performance and descriptions of tree splits

plot(c50) #plot decision tree

varImp(c50) #look at variable importance values

# Combine nicotine and cannabis users into one binary variable to increase power

data$both <- if_else(data$nico > 0 | data$cann > 0, 1, 0)

# Comparing log-normalized cognitive data from nicotine and cannabis users to non-users

## Visual Search Absent Reaction Time - log

## Visual Search Present Reaction Time - log

## Flanker Incongruent Reaction Time - log

## Flanker Congruent Reaction Time - log

### Levene Test

leveneTest(vs_a_rt_log ~ as.factor(both), data.frame(data)) #test for equality of variance

leveneTest(vs_p_rt_log ~ as.factor(both), data.frame(data)) #test for equality of variance

leveneTest(f_i_rt_log ~ as.factor(both), data.frame(data)) #test for equality of variance

leveneTest(f_c_rt_log ~ as.factor(both), data.frame(data)) #test for equality of variance

### ANOVAS for values that meet assumptions

oneway.test(data$vs_a_rt_log ~ data$both, var.equal = TRUE) #test for distribution difference

oneway.test(data$vs_p_rt_log ~ data$both, var.equal = TRUE) #test for distribution difference

oneway.test(data$f_i_rt_log ~ data$both, var.equal = TRUE) #test for distribution difference

oneway.test(data$f_c_rt_log ~ data$both, var.equal = TRUE) #test for distribution difference

### Kruskal for accuracy values that do not meet ANOVA assumptions

kruskal.test(vs_a_acc ~ both, data = data) #test for distribution difference

kruskal.test(vs_p_acc ~ both, data = data) #test for distribution difference

kruskal.test(f_i_acc ~ both, data = data) #test for distribution difference

kruskal.test(f_c_acc ~ both, data = data) #test for distribution difference

# Assessing Trust Scores Between Users and Non-users

leveneTest(score ~ as.factor(both), data.frame(data)) #test for equality of variance

oneway.test(data$score ~ data$both, var.equal = TRUE) #test for distribution difference
