# Supplementary material for: Protocol optimization and reducing dropout in online research
Source: Front Hum Neurosci. 2023 Dec 5;17:1251174. doi: 10.3389/fnhum.2023.1251174 (PMC10729001; doi:10.3389/fnhum.2023.1251174)
Supplement: Supplementary file 3 [file Data_Sheet_2.PDF]

Experiment Workflow in Gorilla Experiment Builder

Design Tab

The study tasks are connected on the *Design tab:* Home → Projects → Experiment → Design. Tasks are added with the “Add node button.”

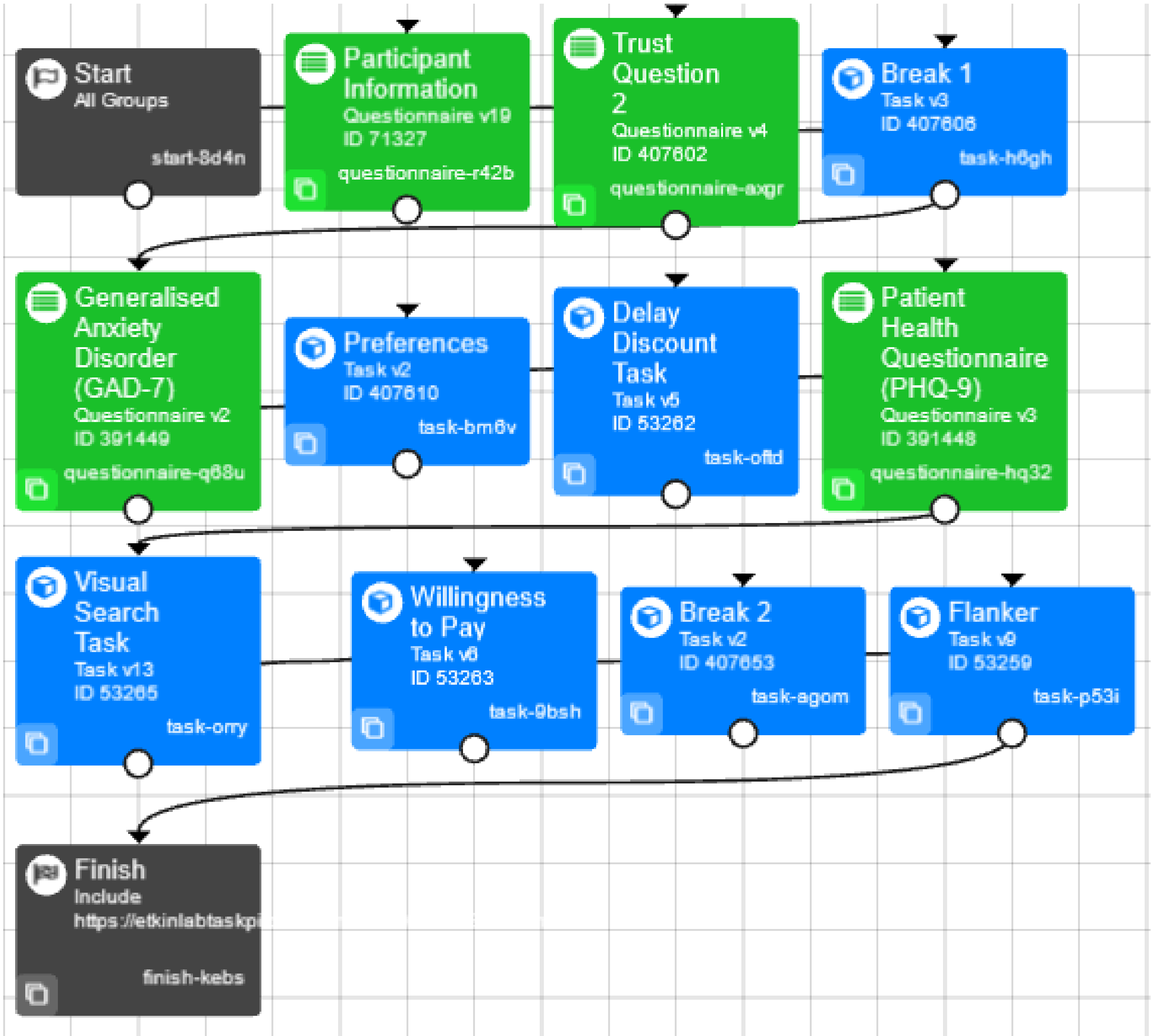

Participant Information Questionnaire Settings in Gorilla Experiment Builder

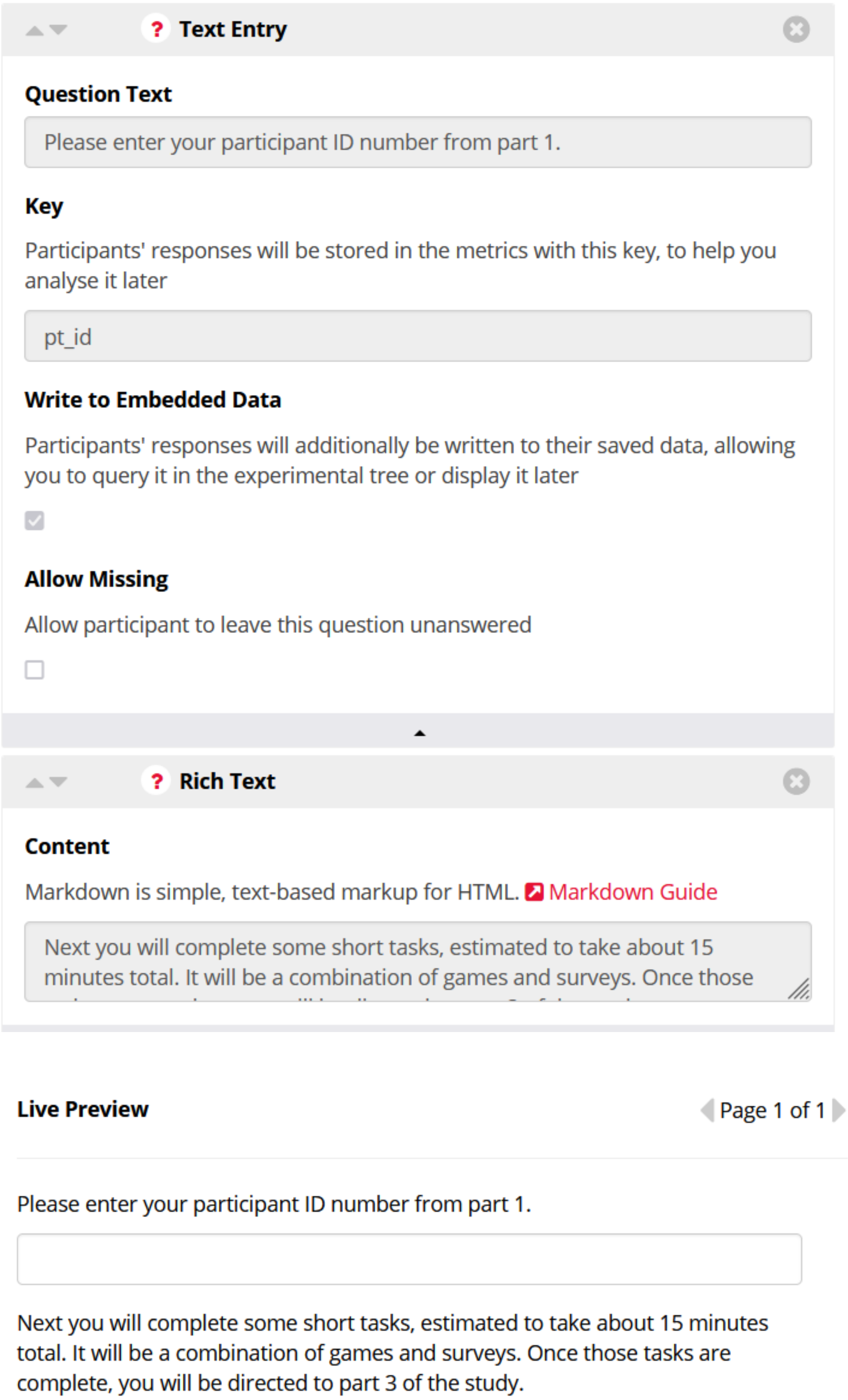

Attention Check Question Settings in Gorilla Experiment Builder

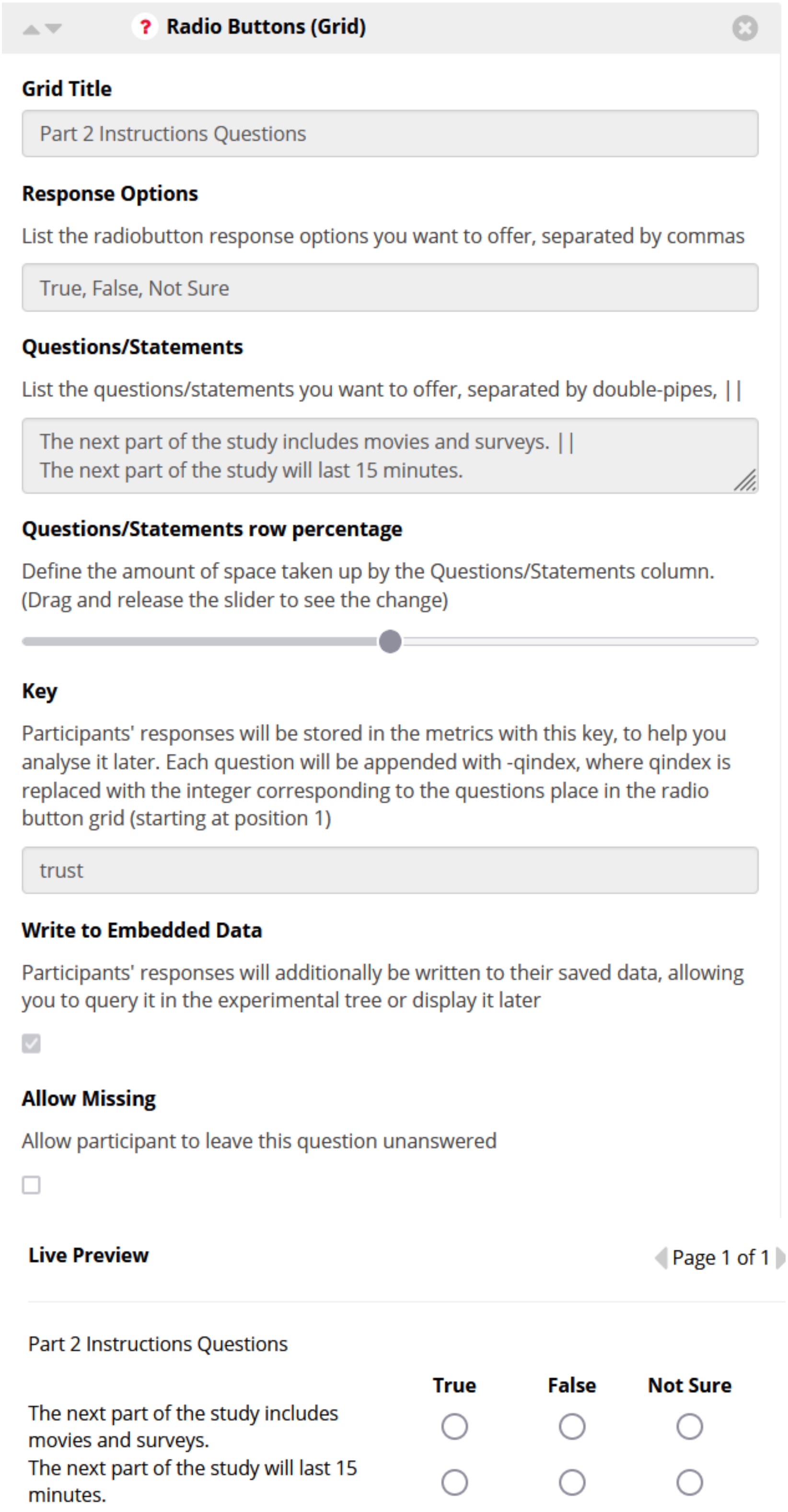

Break Settings in Gorilla Experiment Builder (same for Break 1 and Break 2)

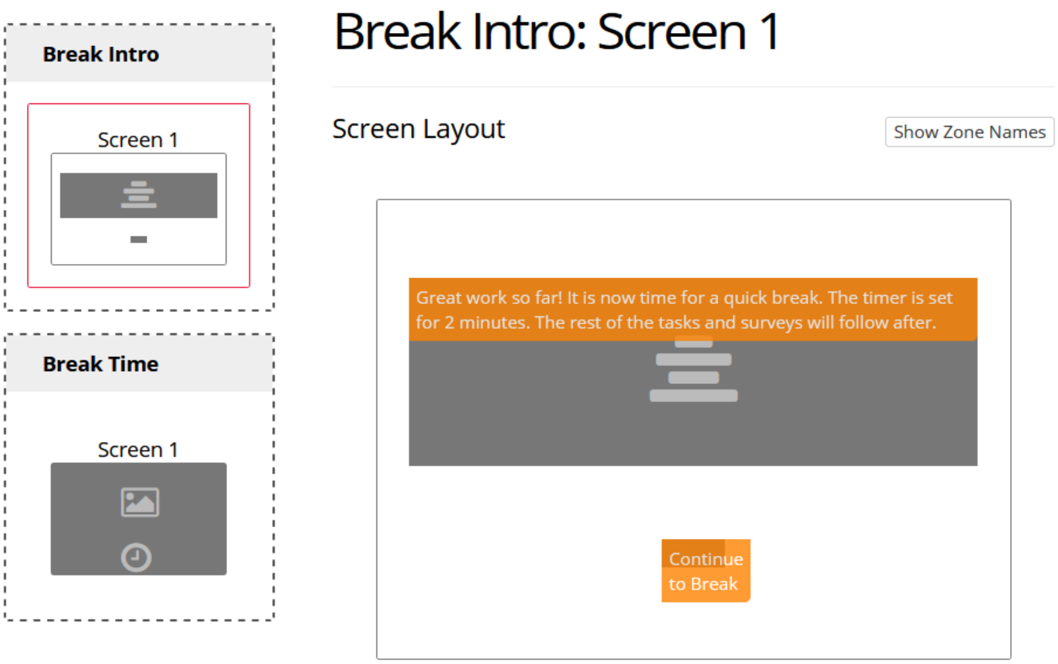

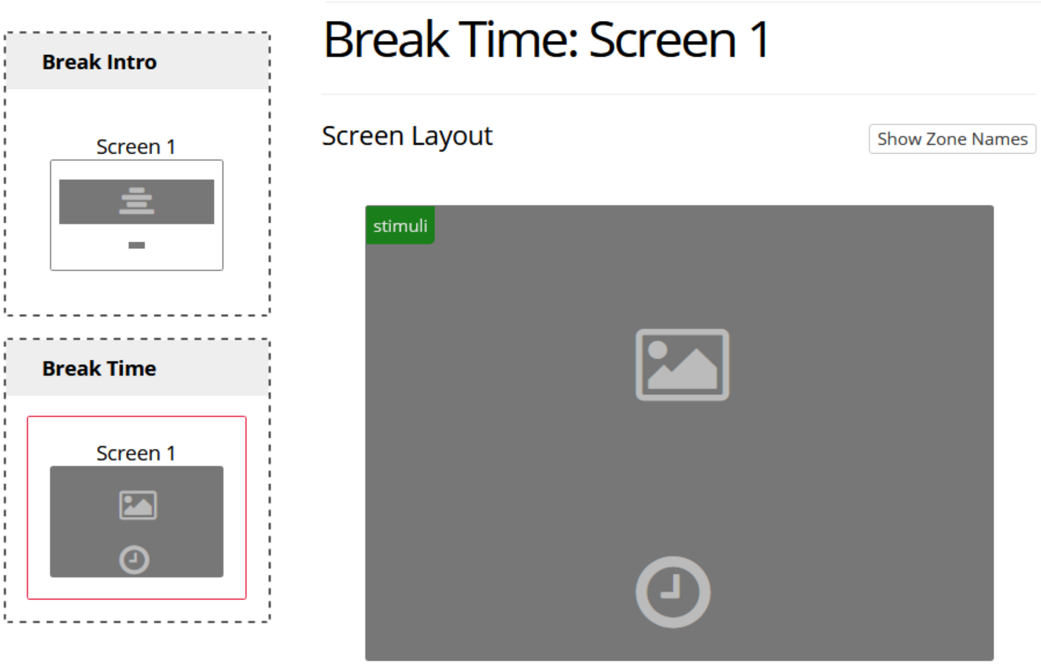

Generalized Anxiety Disorder 7 Settings in Gorilla Experiment Builder

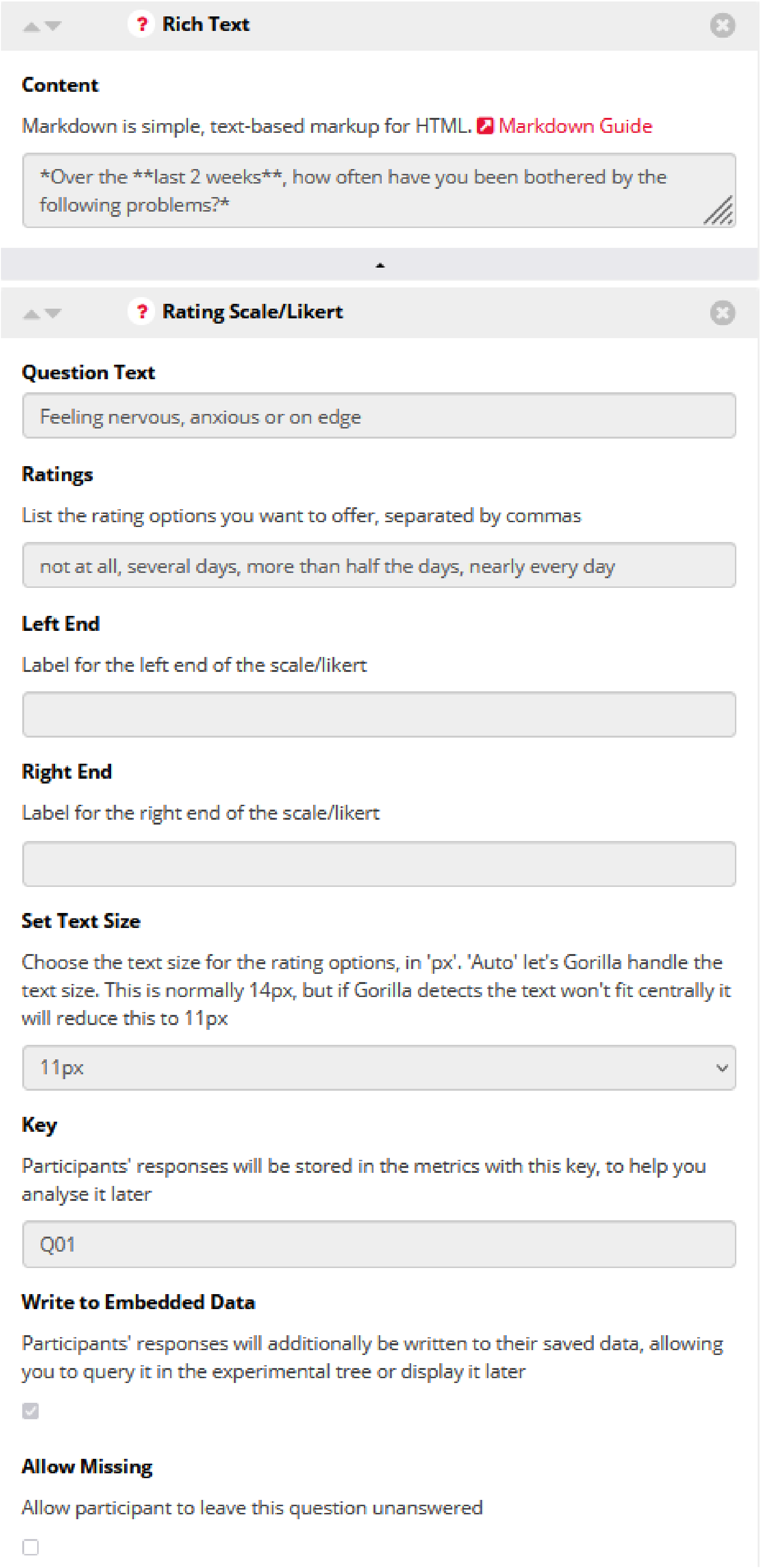

Live Preview Page 1 of 1

*Over the **last 2 weeks**, how often have you been bothered by the following problems?*

Feeling nervous, anxious or on edge

not at all	several days	more than half the days	nearly every day

Not being able to stop or control worrying

not at all	several days	more than half the days	nearly every day

Worrying too much about different things

not at all	several days	more than half the days	nearly every day

Trouble relaxing

not at all	several days	more than half the days	nearly every day

Being so restless that it is hard to sit still

not at all	several days	more than half the days	nearly every day

Becoming easily annoyed or irritable

not at all	several days	more than half the days	nearly every day

Feeling afraid as if something awful might happen

not at all	several days	more than half the days	nearly every day

Travel Preferences Survey Settings in Gorilla Experiment Builder

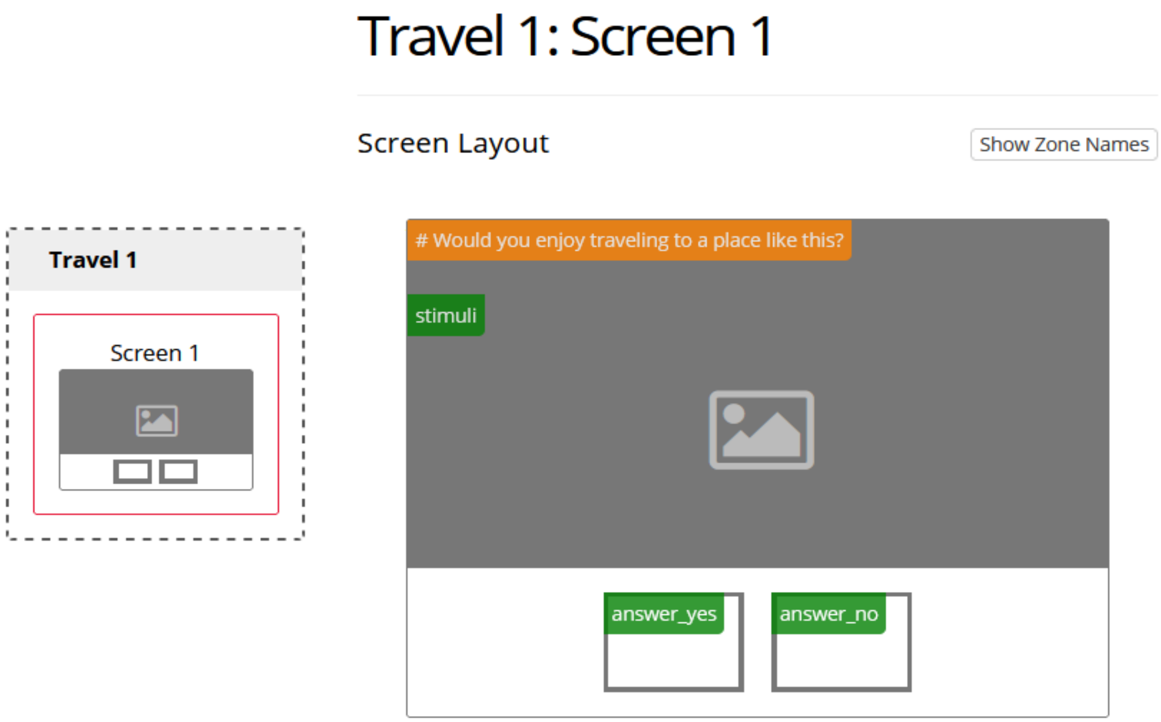

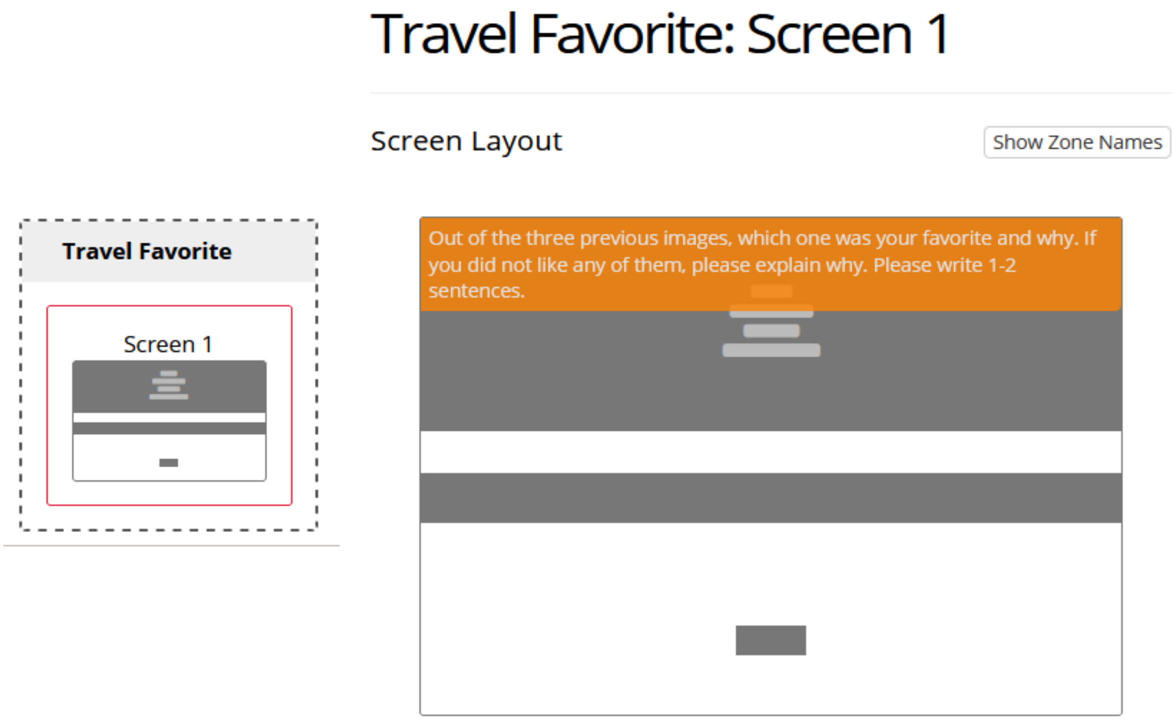

Delay Discounting Settings in Gorilla Experiment Builder

Instructions

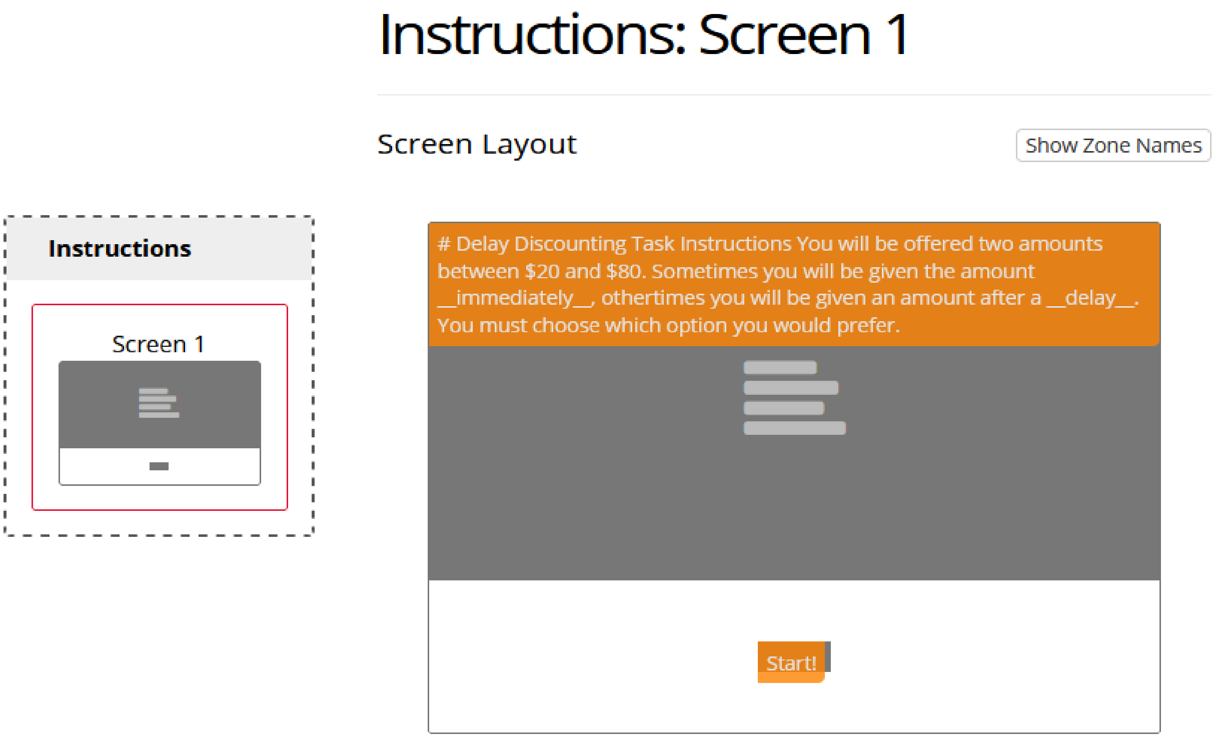

Trials

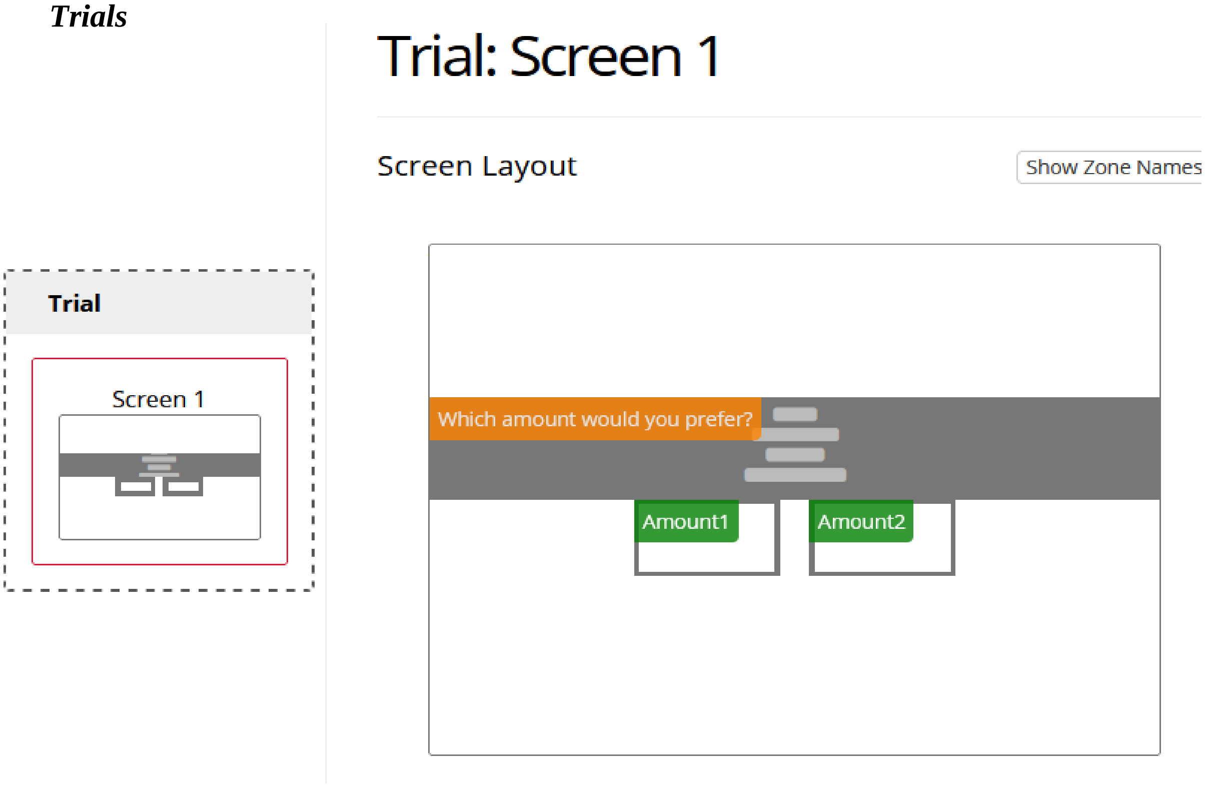

Debrief

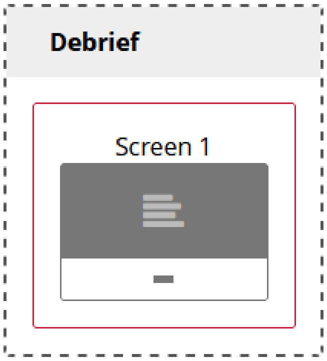
Debrief
Screen 1

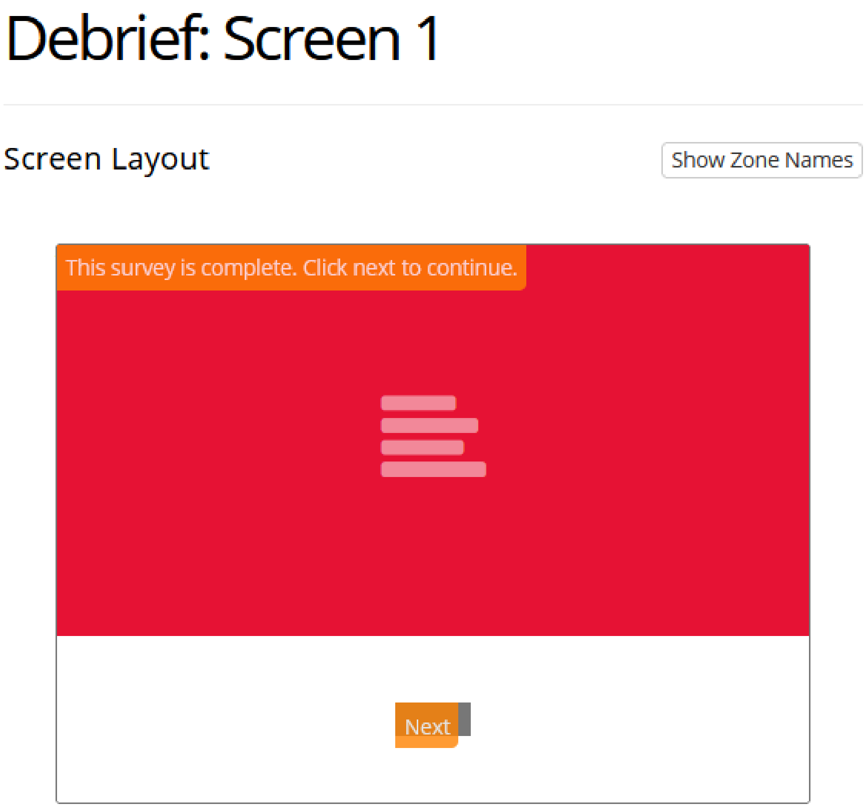
Debrief: Screen 1
Screen Layout
Show Zone Names
This survey is complete. Click next to continue.
Next

Spreadsheet with Delayed Discounting Trial Settings

Row	randomise_blocks	randomise_trials	display	Amount1	Amount2
1			Instructions		
2		1	Trial	Receive $54 today	Receive $55 in 117 days
3		1	Trial	Receive $55 today	Receive $75 in 61 days
4		1	Trial	Receive $19 today	Receive $25 in 53 days
5		1	Trial	Receive $31 today	Receive $85 in 7 days
6		1	Trial	Receive $14 today	Receive $25 in 19 days
7		1	Trial	Receive $47 today	Receive $50 in 160 days
8		1	Trial	Receive $15 today	Receive $35 in 13 days
9		1	Trial	Receive $25 today	Receive $60 in 14 days
10		1	Trial	Receive $78 today	Receive $80 in 162 days
11		1	Trial	Receive $40 today	Receive $55 in 62 days
12		1	Trial	Receive $11 today	Receive $30 in 7 days
13		1	Trial	Receive $67 today	Receive $75 in 119 days
14		1	Trial	Receive $34 today	Receive $35 in 186 days
15		1	Trial	Receive $27 today	Receive $50 in 21 days
16		1	Trial	Receive $69 today	Receive $85 in 91 days
17		1	Trial	Receive $49 today	Receive $60 in 89 days
18		1	Trial	Receive $80 today	Receive $85 in 157 days
19		1	Trial	Receive $24 today	Receive $35 in 29 days
20		1	Trial	Receive $33 today	Receive $80 in 14 days
21		1	Trial	Receive $28 today	Receive $30 in 179 days
22		1	Trial	Receive $34 today	Receive $50 in 30 days
23		1	Trial	Receive $25 today	Receive $30 in 80 days
24		1	Trial	Receive $41 today	Receive $75 in 20 days
25		1	Trial	Receive $54 today	Receive $60 in 111 days
26		1	Trial	Receive $54 today	Receive $80 in 30 days
27		1	Trial	Receive $22 today	Receive $25 in 136 days
28		1	Trial	Receive $20 today	Receive $55 in 7 days
29			Debrief		

Patient Health Questionnaire 8 Settings in Gorilla Experiment Builder

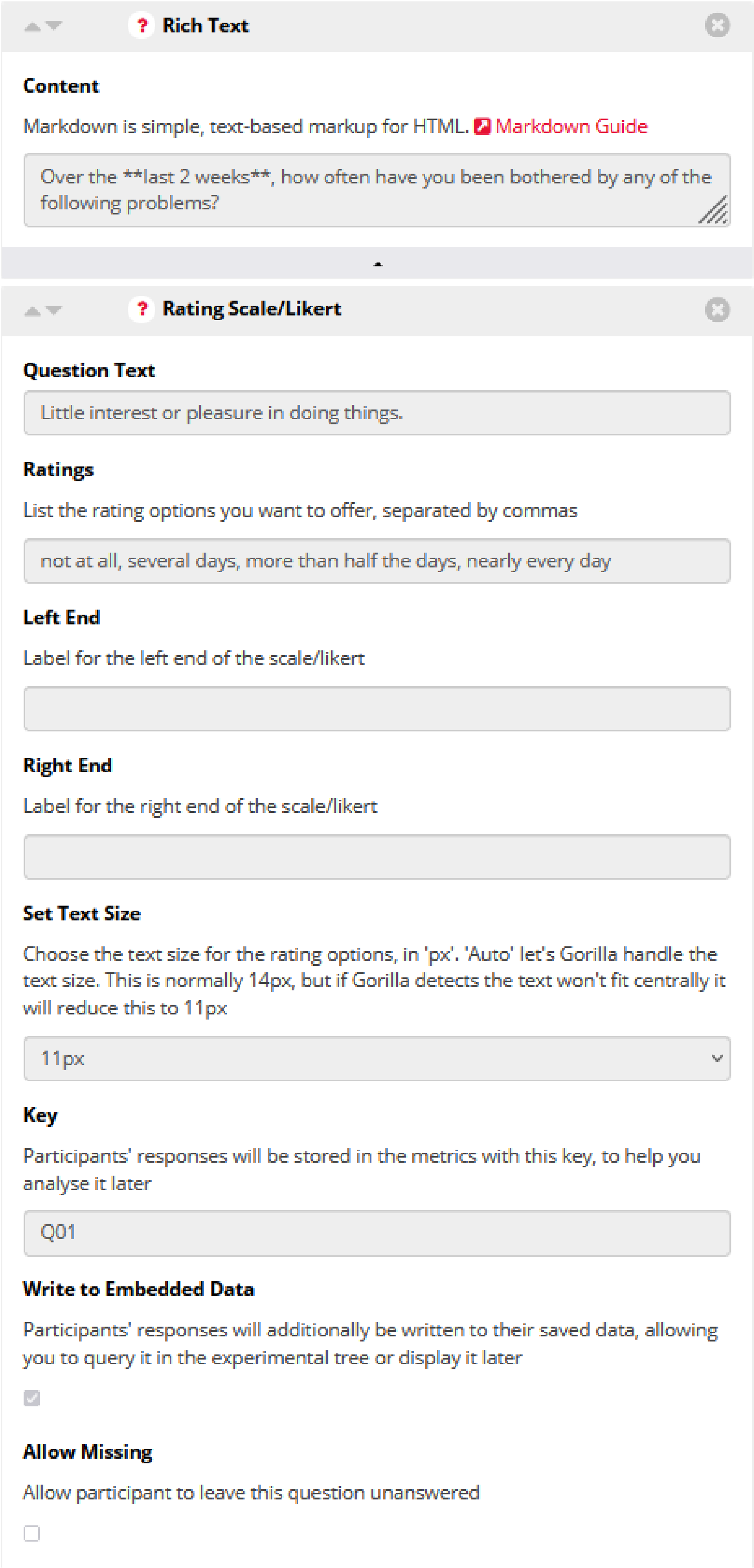

Live Preview

Over the **last 2 weeks**, how often have you been bothered by any of the following problems?

Little interest or pleasure in doing things.

not at all	several days	more than half the days	nearly every day

Feeling down, depressed, or hopeless.

not at all	several days	more than half the days	nearly every day

Trouble falling or staying asleep, or sleeping too much.

not at all	several days	more than half the days	nearly every day

Feeling tired or having little energy.

not at all	several days	more than half the days	nearly every day

Poor appetite or overeating.

not at all	several days	more than half the days	nearly every day

Feeling bad about yourself - or that you are a failure or have let yourself or your family down.

not at all	several days	more than half the days	nearly every day

Trouble concentrating on things, such as reading the newspaper or watching television.

not at all	several days	more than half the days	nearly every day

Moving or speaking so slowly that other people could have noticed? Or the opposite - being so fidgety or restless that you have been moving around a lot more than usual.

not at all	several days	more than half the days	nearly every day

Visual Search Task Settings in Gorilla Experiment Builder

Instructions

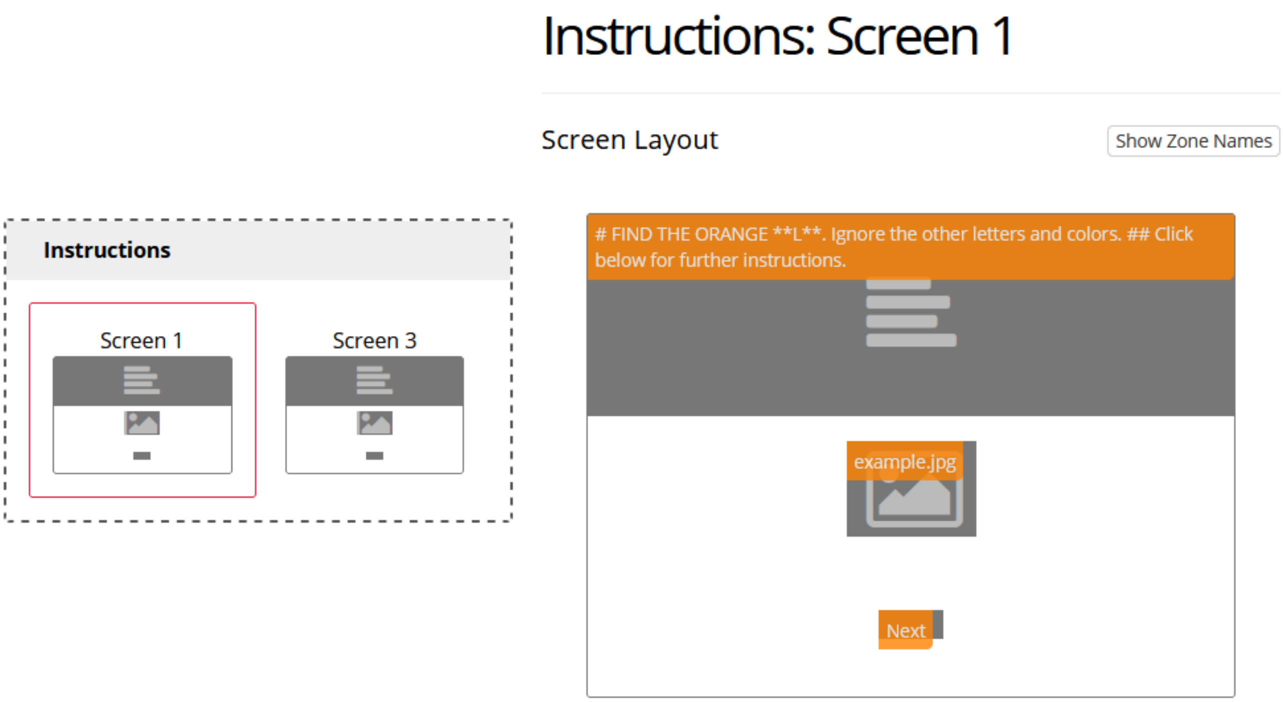

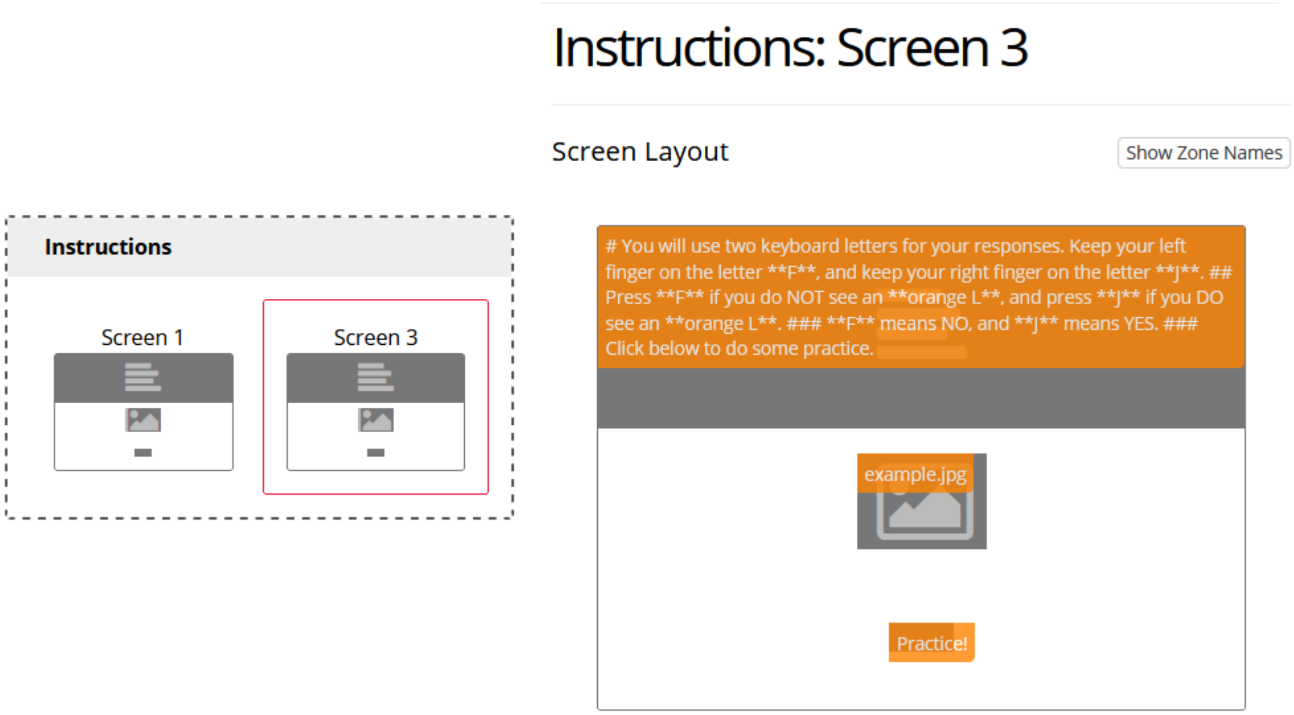

Practice Trials

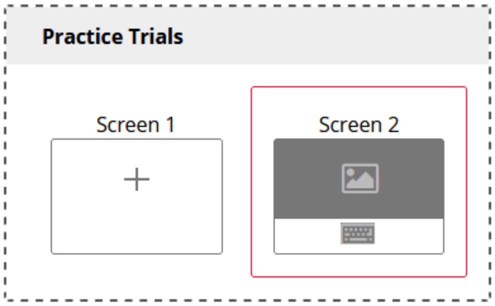

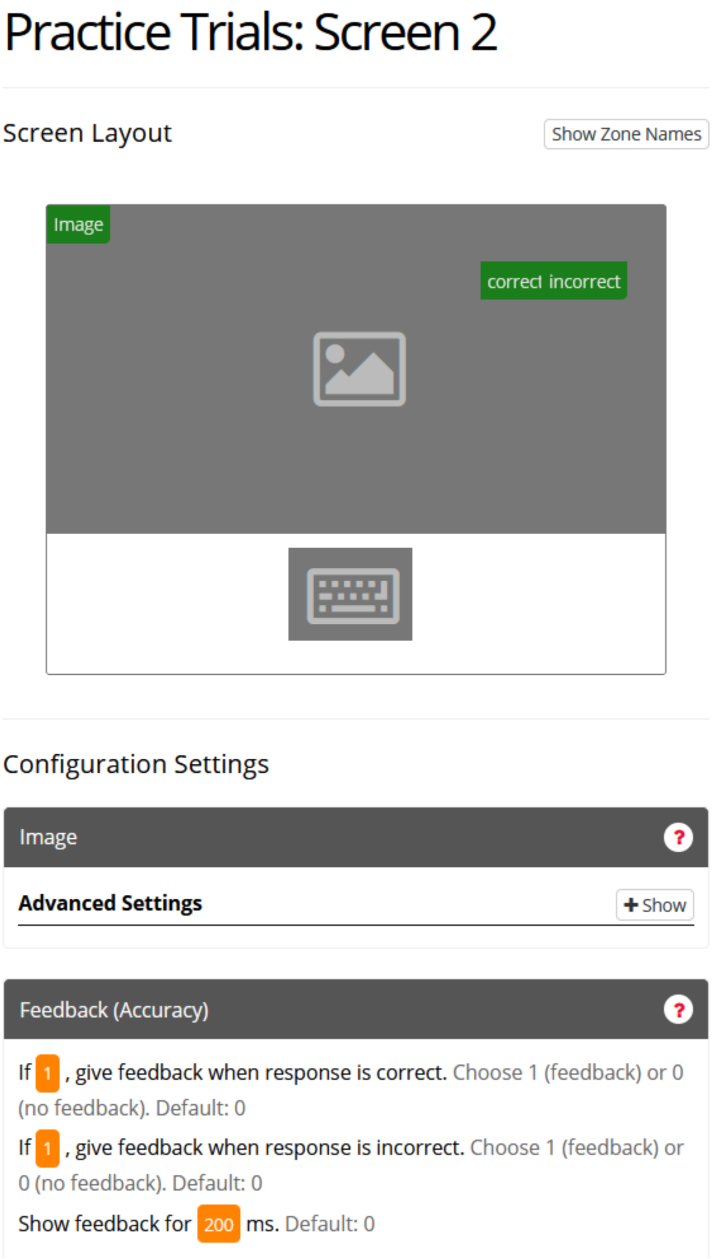

Trials

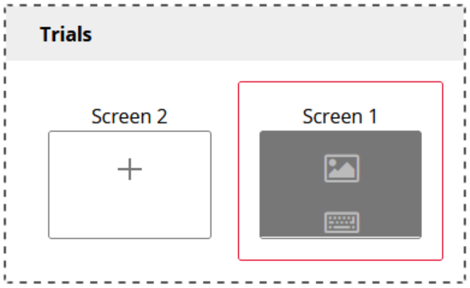
Trials
Screen 2
Screen 1

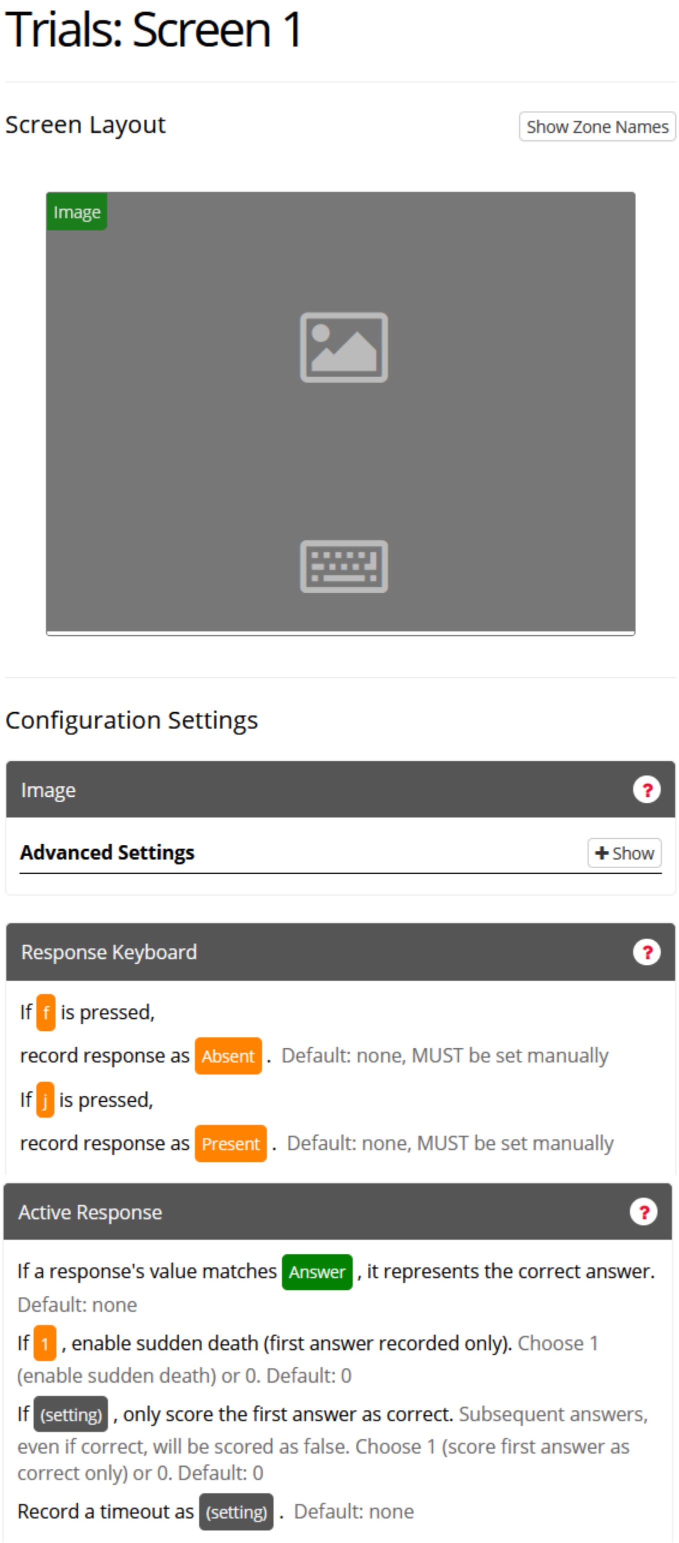
Trials: Screen 1
Screen Layout
Show Zone Names
Image
Configuration Settings
Image
Advanced Settings
Show
Response Keyboard
If f is pressed,
record response as Absent . Default: none, MUST be set manually
If j is pressed,
record response as Present . Default: none, MUST be set manually
Active Response
If a response's value matches Answer , it represents the correct answer. Default: none
If 1 , enable sudden death (first answer recorded only). Choose 1 (enable sudden death) or 0. Default: 0
If (setting) , only score the first answer as correct. Subsequent answers, even if correct, will be scored as false. Choose 1 (score first answer as correct only) or 0. Default: 0
Record a timeout as (setting) . Default: none

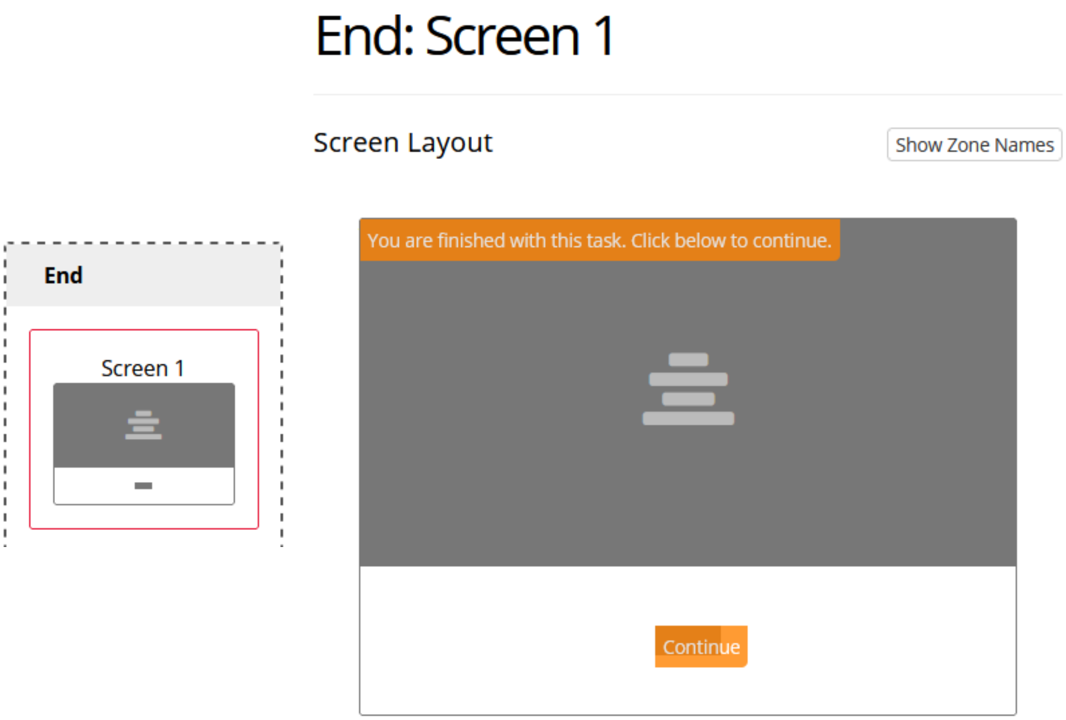

Feedback Stimuli and Array Stimuli

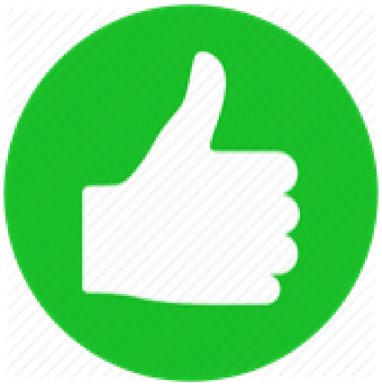

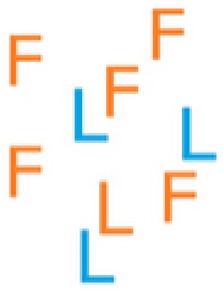

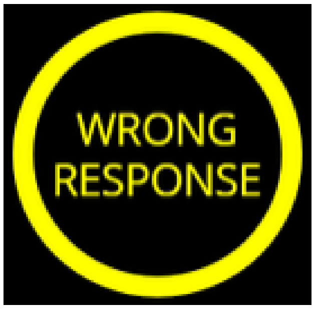

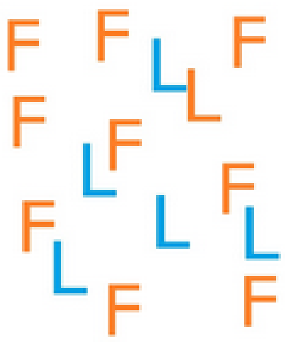

Spreadsheet of Visual Search Trial Settings

Row	randomise_blocks	randomise_trials	display	Image	Answer	correct	incorrect
1			Instructions				
2			Practice Trials	Practice1_crop.jpg	Present	good.png	WrongResponse.png
3			Practice Trials	Practice2_crop.jpg	Present	good.png	WrongResponse.png
4			Practice Trials	Practice3_crop.jpg	Absent	good.png	WrongResponse.png
5			Real Start				
6		1	Trials	3Image15.jpg	Present	good.png	WrongResponse.png
7		1	Trials	4ImageNoTarget.jpg	Absent	good.png	WrongResponse.png
8		1	Trials	4Image8.jpg	Absent	good.png	WrongResponse.png
9		1	Trials	1Image8.jpg	Present	good.png	WrongResponse.png
10		1	Trials	1Image15.jpg	Present	good.png	WrongResponse.png
11		1	Trials	2ImageNT.jpg	Absent	good.png	WrongResponse.png
12		1	Trials	3Image8.jpg	Present	good.png	WrongResponse.png
13		1	Trials	2Image15.jpg	Present	good.png	WrongResponse.png
14		1	Trials	3Image15.jpg	Present	good.png	WrongResponse.png
15		1	Trials	4ImageNoTarget.jpg	Absent	good.png	WrongResponse.png
16		1	Trials	4Image8.jpg	Absent	good.png	WrongResponse.png
17		1	Trials	1Image8.jpg	Present	good.png	WrongResponse.png
18		1	Trials	1Image15.jpg	Present	good.png	WrongResponse.png
19		1	Trials	2ImageNT.jpg	Absent	good.png	WrongResponse.png
20		1	Trials	3Image8.jpg	Present	good.png	WrongResponse.png
21		1	Trials	2Image15.jpg	Present	good.png	WrongResponse.png
22		1	Trials	3Image15.jpg	Present	good.png	WrongResponse.png
23		1	Trials	4ImageNoTarget.jpg	Absent	good.png	WrongResponse.png
24		1	Trials	4Image8.jpg	Absent	good.png	WrongResponse.png
25		1	Trials	1Image8.jpg	Present	good.png	WrongResponse.png
26		1	Trials	1Image15.jpg	Present	good.png	WrongResponse.png
27		1	Trials	2ImageNT.jpg	Absent	good.png	WrongResponse.png
28		1	Trials	3Image8.jpg	Present	good.png	WrongResponse.png
29		1	Trials	2Image15.jpg	Present	good.png	WrongResponse.png
30			End				

Willingness to Pay Task Settings in Gorilla Experiment Builder

Instructions

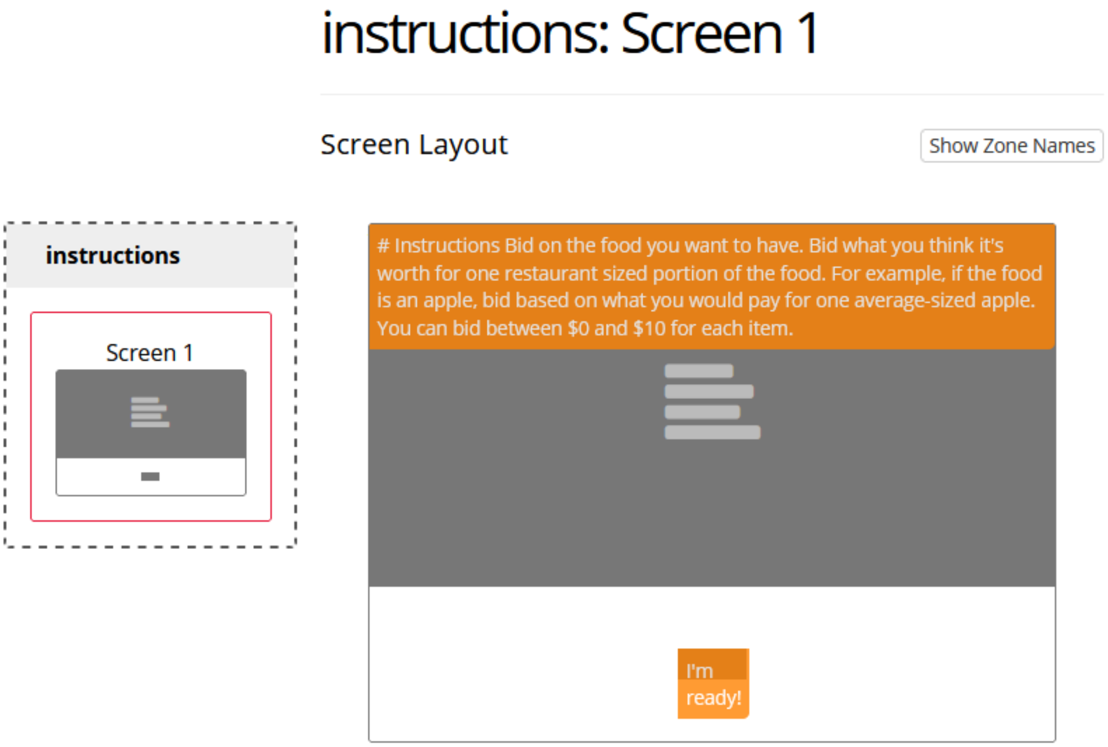

Trials

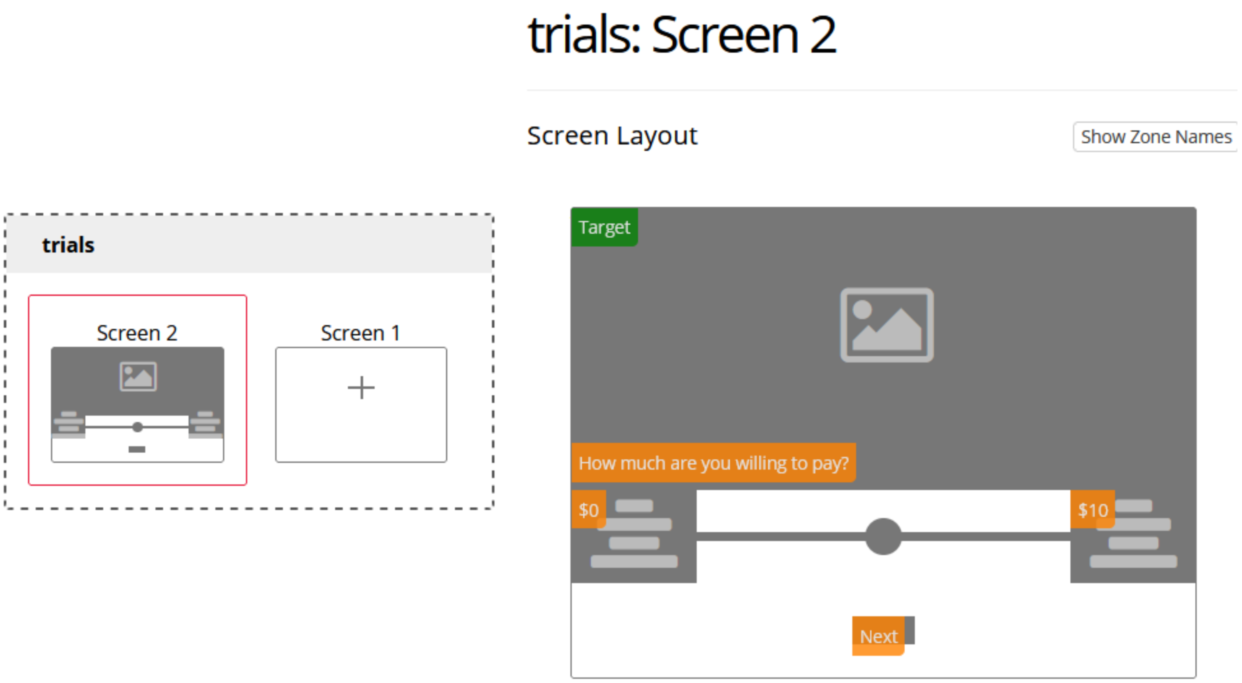

Debrief

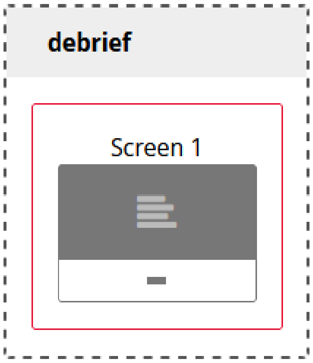

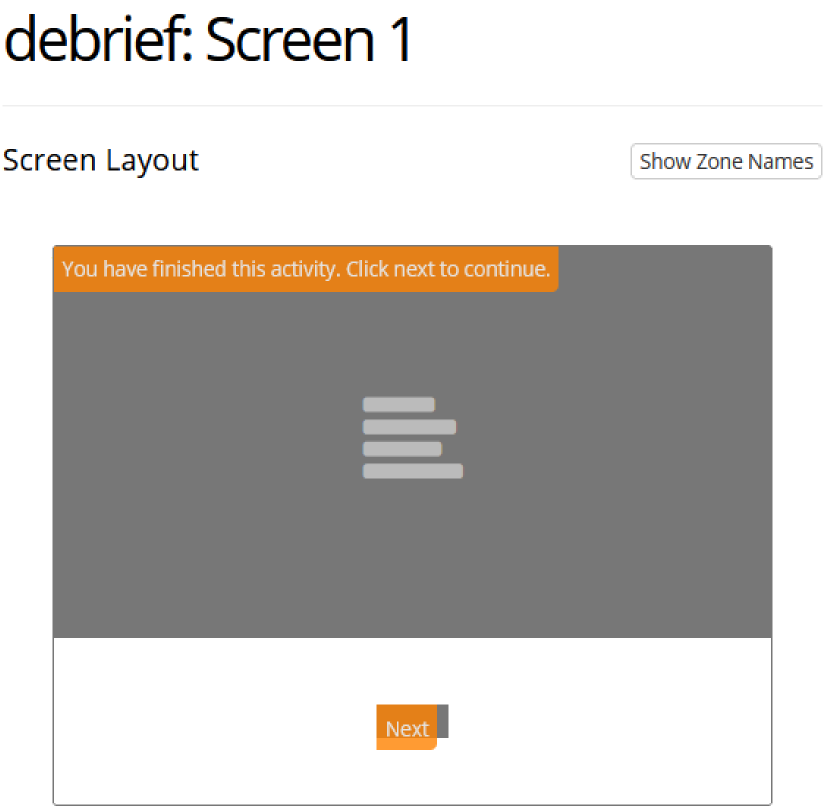

Stimuli

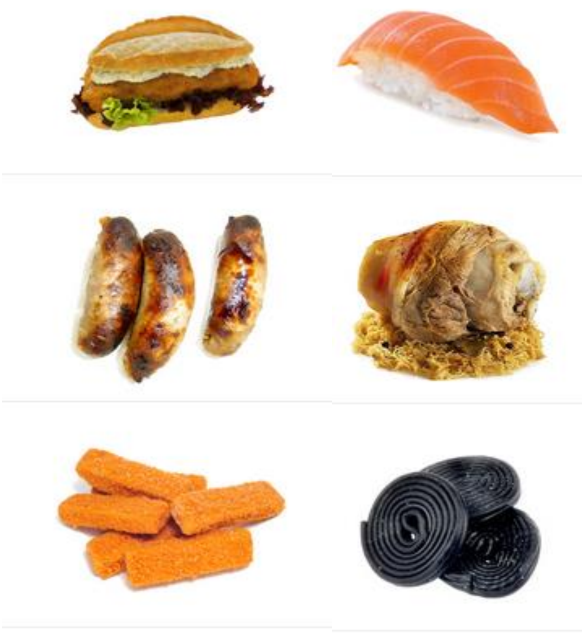

Spreadsheet for Willingness to Pay Trial Settings

Row	randomise_blocks	randomise_trials	display	Target
1			instructions	
2		1	trials	0069.jpg
3		1	trials	0191.jpg
4		1	trials	0302.jpg
5		1	trials	0308.jpg
6		1	trials	0322.jpg
7		1	trials	0336.jpg
8		1	trials	0408.jpg
9		1	trials	0438.jpg
10		1	trials	0446.jpg
11		1	trials	0465.jpg
12		1	trials	0466.jpg
13		1	trials	0467.jpg
14		1	trials	0469.jpg
15		1	trials	0473.jpg
16		1	trials	0474.jpg
17		1	trials	0476.jpg
18		1	trials	0477.jpg
19		1	trials	0478.jpg
20		1	trials	0480.jpg
21		1	trials	0483.jpg
22		1	trials	0488.jpg
23		1	trials	0489.jpg
24		1	trials	0491.jpg
25		1	trials	0493.jpg
26		1	trials	0494.jpg
27		1	trials	0497.jpg
28		1	trials	0504.jpg
…				
57		1	trials	0566.jpg
58			debrief	

Flanker Task Settings in Gorilla Experiment Builder

Instructions

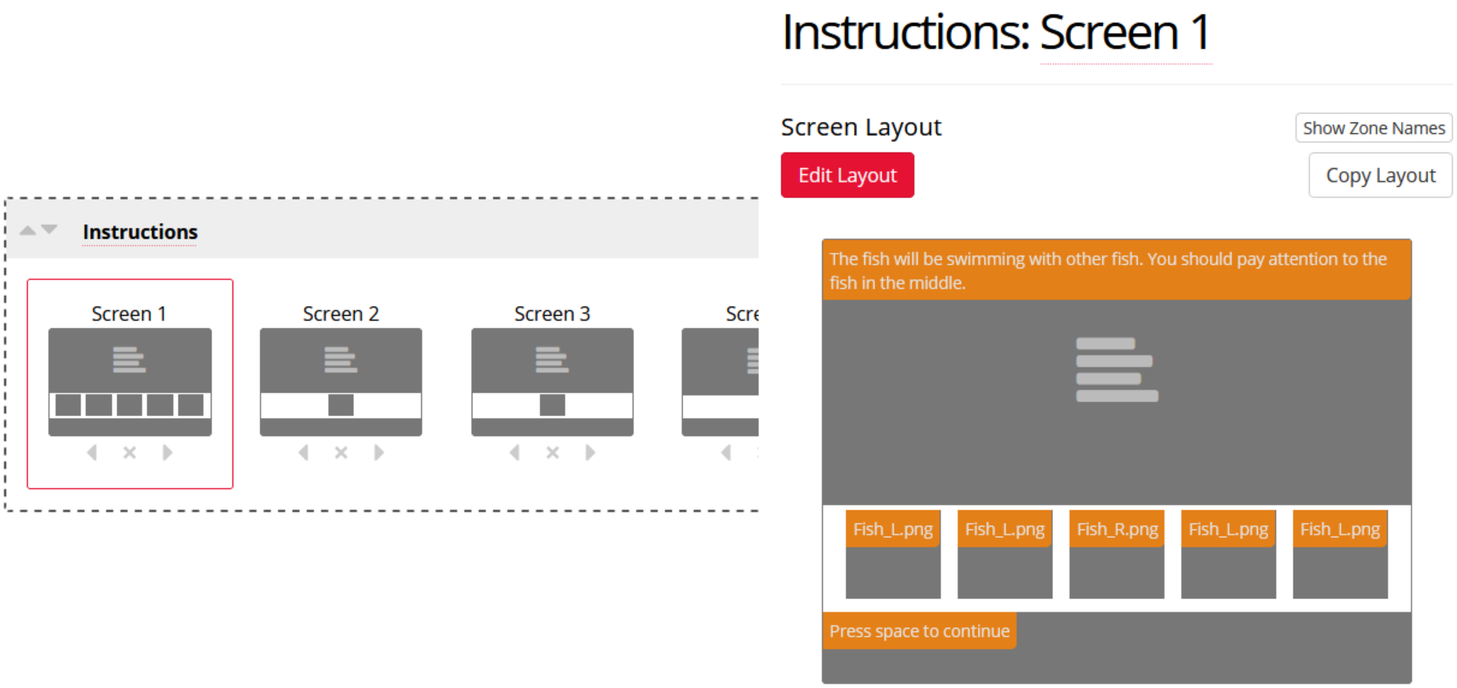

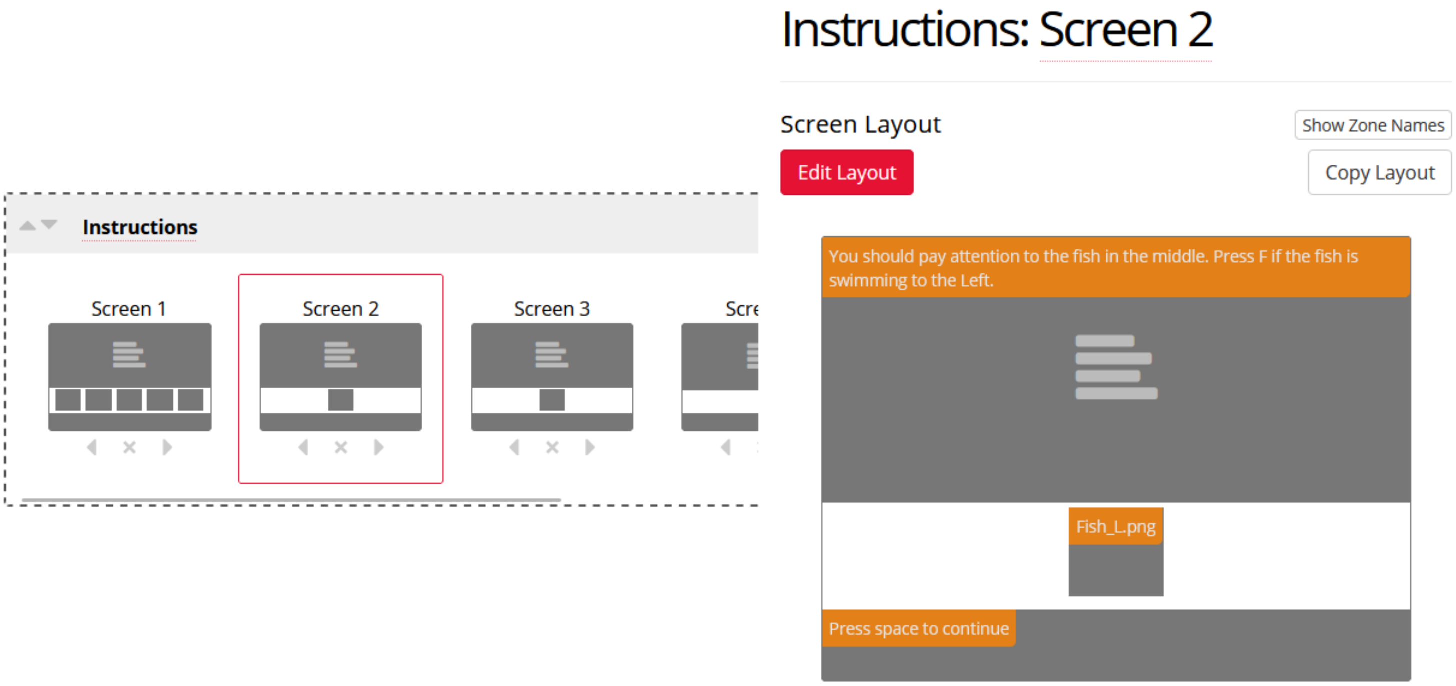

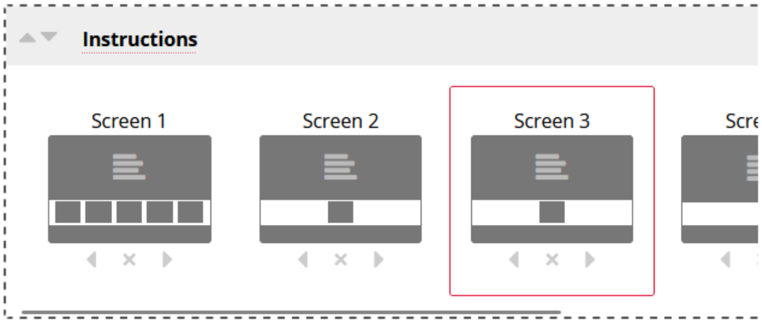
Instructions
Screen 1
Screen 2
Screen 3

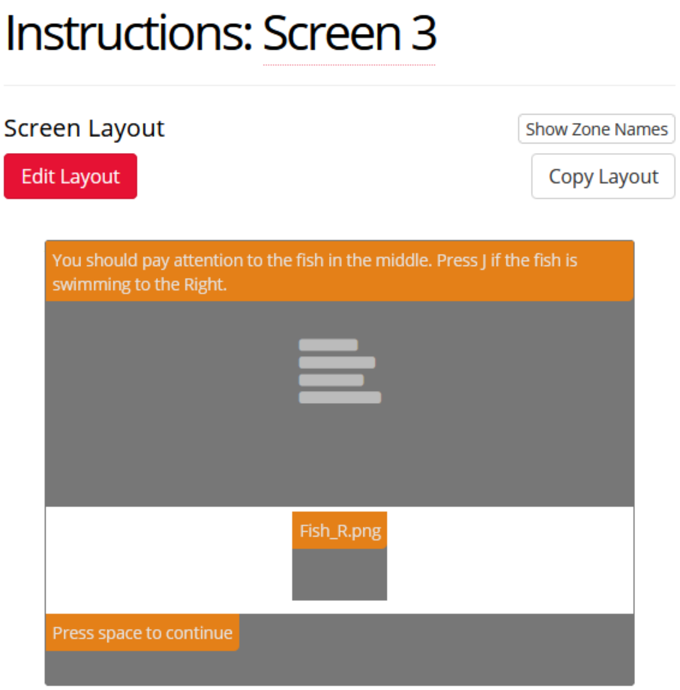
Instructions: Screen 3
Screen Layout
Show Zone Names
Edit Layout
Copy Layout
You should pay attention to the fish in the middle. Press J if the fish is swimming to the Right.
Fish_R.png
Press space to continue

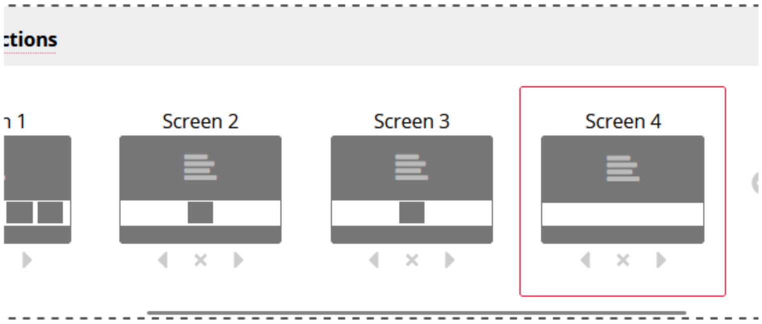
Screen 2
Screen 3
Screen 4

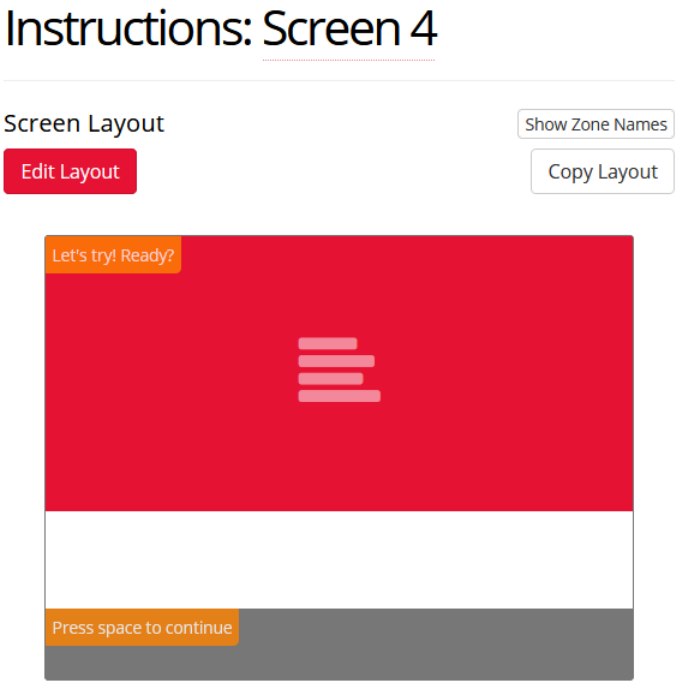
Instructions: Screen 4
Screen Layout
Show Zone Names
Edit Layout
Copy Layout
Let's try! Ready?
Press space to continue

Trials

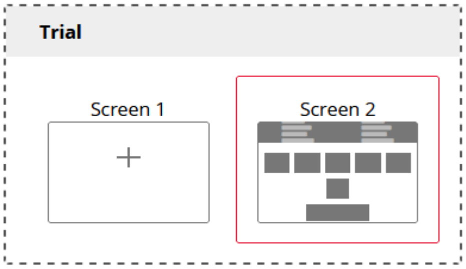
Trial
Screen 1
Screen 2

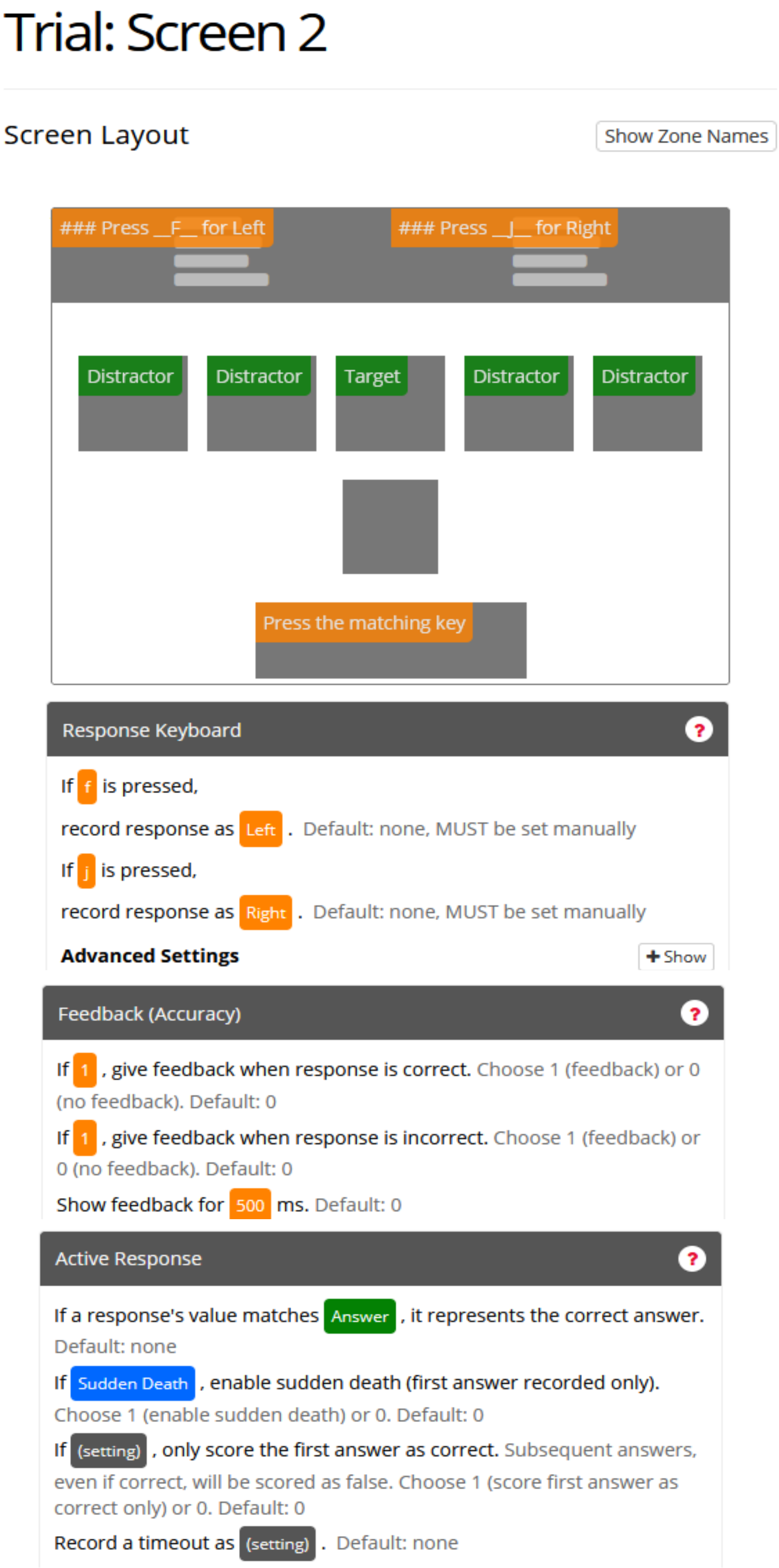
Trial: Screen 2
Screen Layout
Show Zone Names
Press __F__ for Left
Press __J__ for Right
Distractor
Distractor
Target
Distractor
Distractor
Press the matching key
Response Keyboard
If f is pressed,
record response as Left . Default: none, MUST be set manually
If j is pressed,
record response as Right . Default: none, MUST be set manually
Advanced Settings
+ Show
Feedback (Accuracy)
If 1 , give feedback when response is correct. Choose 1 (feedback) or 0 (no feedback). Default: 0
If 1 , give feedback when response is incorrect. Choose 1 (feedback) or 0 (no feedback). Default: 0
Show feedback for 500 ms. Default: 0
Active Response
If a response's value matches Answer , it represents the correct answer. Default: none
If Sudden Death , enable sudden death (first answer recorded only). Choose 1 (enable sudden death) or 0. Default: 0
If (setting) , only score the first answer as correct. Subsequent answers, even if correct, will be scored as false. Choose 1 (score first answer as correct only) or 0. Default: 0
Record a timeout as (setting) . Default: none

Debrief

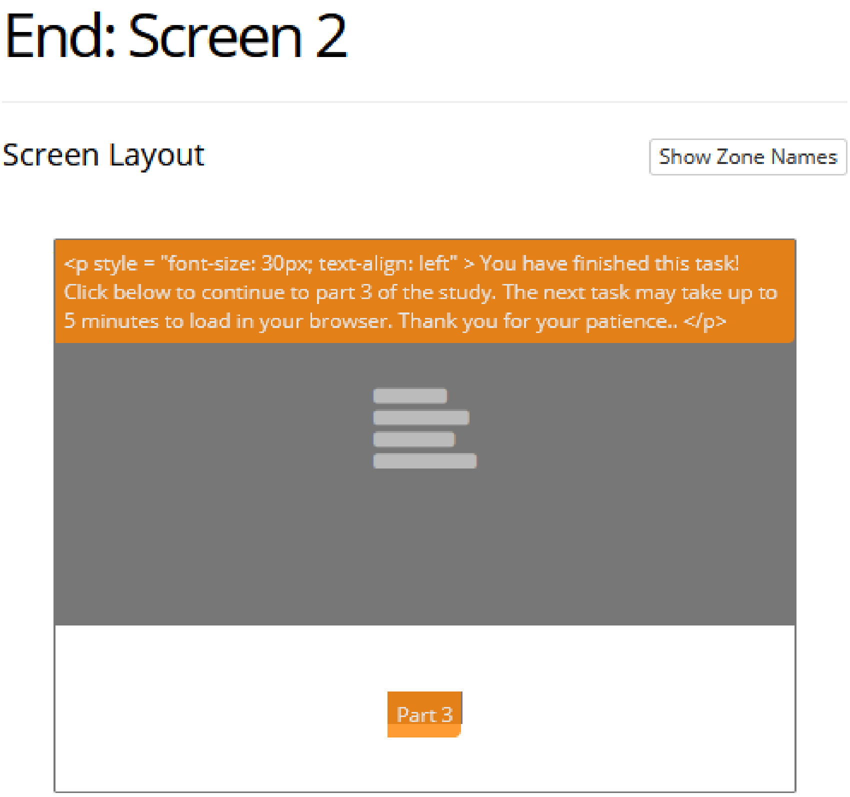

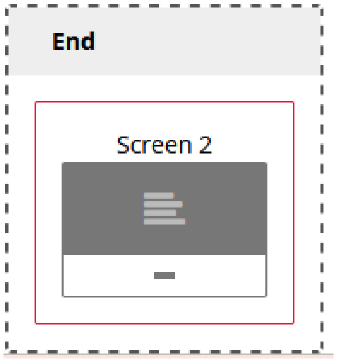

Stimuli

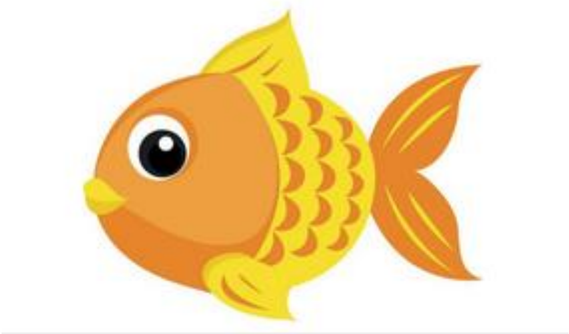

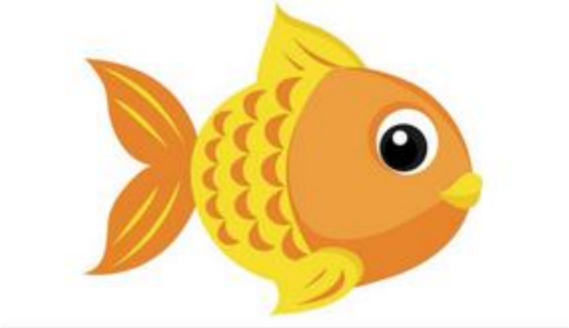

Spreadsheet for Flanker Trial Settings

Row	randomise_blocks	randomise_trials	display	Answer	Target	Distractor	Fixation	Break	totaltrials	correcttrials	Type
1			Instructions								
2		1	Trial	Left	Fish_L.png	Fish_L.png	500				
3		1	Trial	Right	Fish_R.png	Fish_R.png	500				
4		1	Trial	Left	Fish_L.png	Fish_R.png	500				
5		1	Trial	Right	Fish_R.png	Fish_L.png	500				
6			Break					#### Now you've practiced lets have a go for real now! #### Ready?			
7		2	Trial	Right	Fish_R.png	Fish_L.png	1200		total	correct	Incongruent
8		2	Trial	Left	Fish_L.png	Fish_L.png	1200		total	correct	Congruent
9		2	Trial	Right	Fish_R.png	Fish_L.png	1200		total	correct	Incongruent
10		2	Trial	Right	Fish_R.png	Fish_R.png	1200		total	correct	Congruent
11		2	Trial	Right	Fish_R.png	Fish_L.png	800		total	correct	Incongruent
12		2	Trial	Right	Fish_R.png	Fish_L.png	400		total	correct	Incongruent
13		2	Trial	Left	Fish_L.png	Fish_L.png	800		total	correct	Congruent
14		2	Trial	Left	Fish_L.png	Fish_R.png	800		total	correct	Incongruent
15		2	Trial	Left	Fish_L.png	Fish_R.png	1200		total	correct	Incongruent
16		2	Trial	Left	Fish_L.png	Fish_R.png	400		total	correct	Incongruent
17		2	Trial	Right	Fish_R.png	Fish_R.png	400		total	correct	Congruent
18		2	Trial	Right	Fish_R.png	Fish_R.png	800		total	correct	Congruent
19		2	Trial	Right	Fish_R.png	Fish_L.png	1200		total	correct	Incongruent
20		2	Trial	Left	Fish_L.png	Fish_L.png	400		total	correct	Congruent
…											
54		2	Trial	Left	Fish_L.png	Fish_L.png	1200		total	correct	Congruent
55			End								
