# Supplementary material for: Protocol optimization and reducing dropout in online research
Source: Front Hum Neurosci. 2023 Dec 5;17:1251174. doi: 10.3389/fnhum.2023.1251174 (PMC10729001; doi:10.3389/fnhum.2023.1251174)

## Unity Task Settings

### *Rating Food Stimuli*

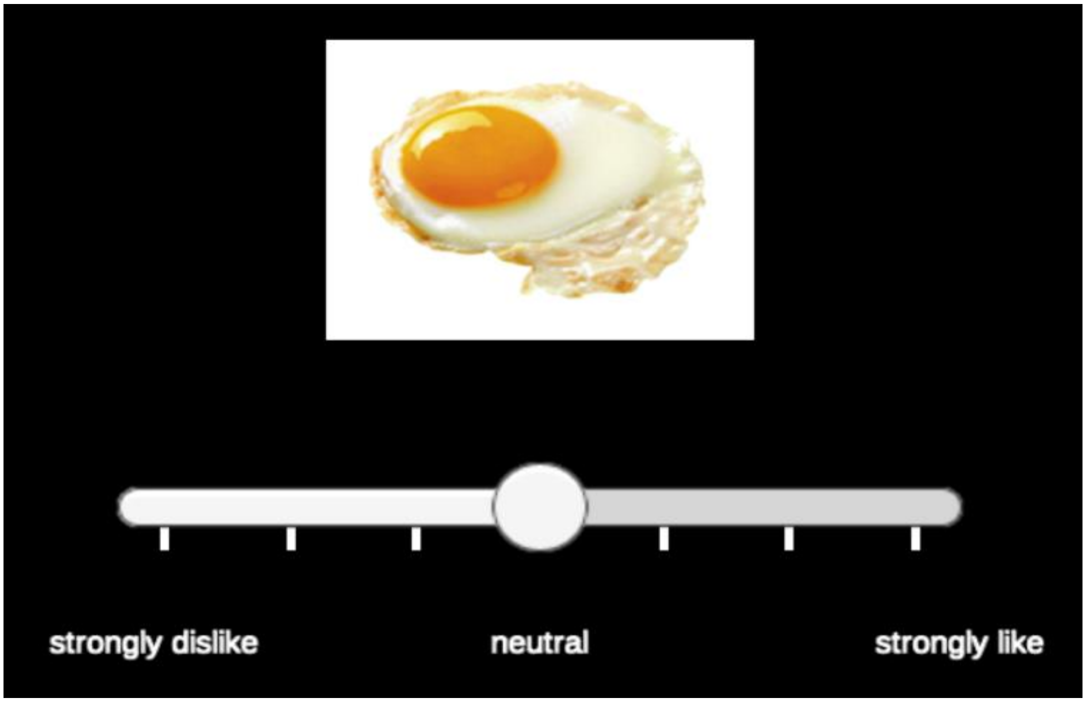


### *Instructions for Grid Search Buttons*

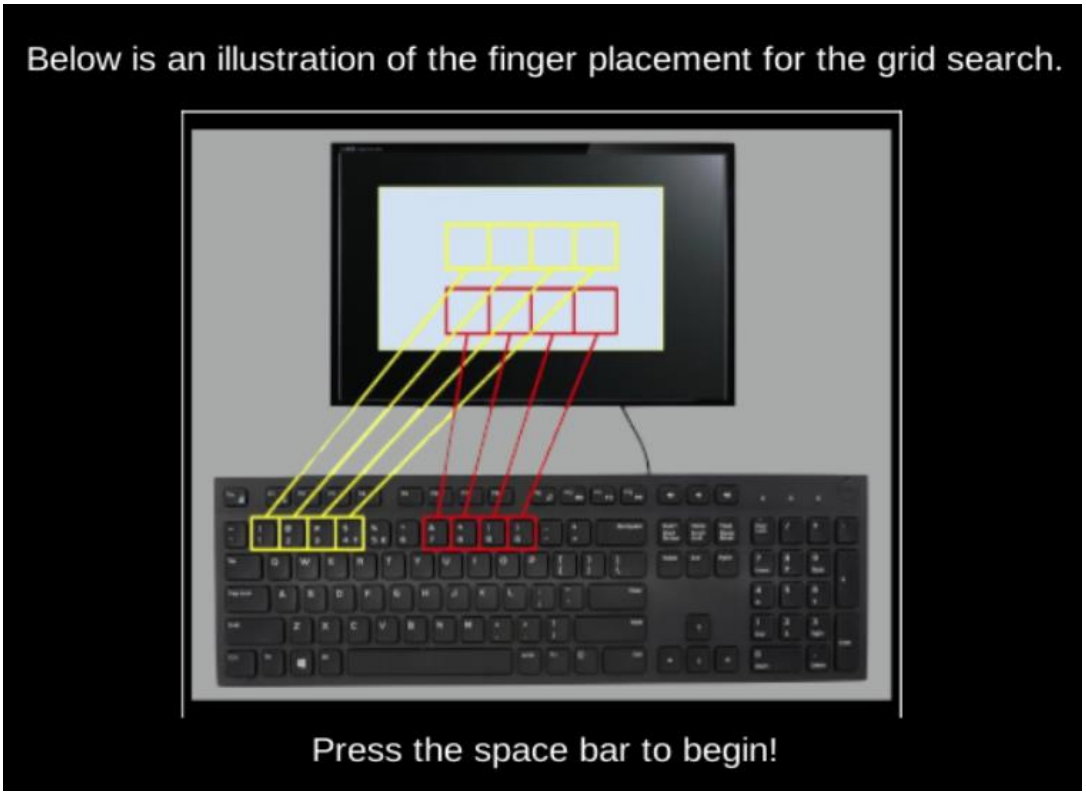

***Arrays of 3-7 Distractors with Neutral Target (clipboard)***

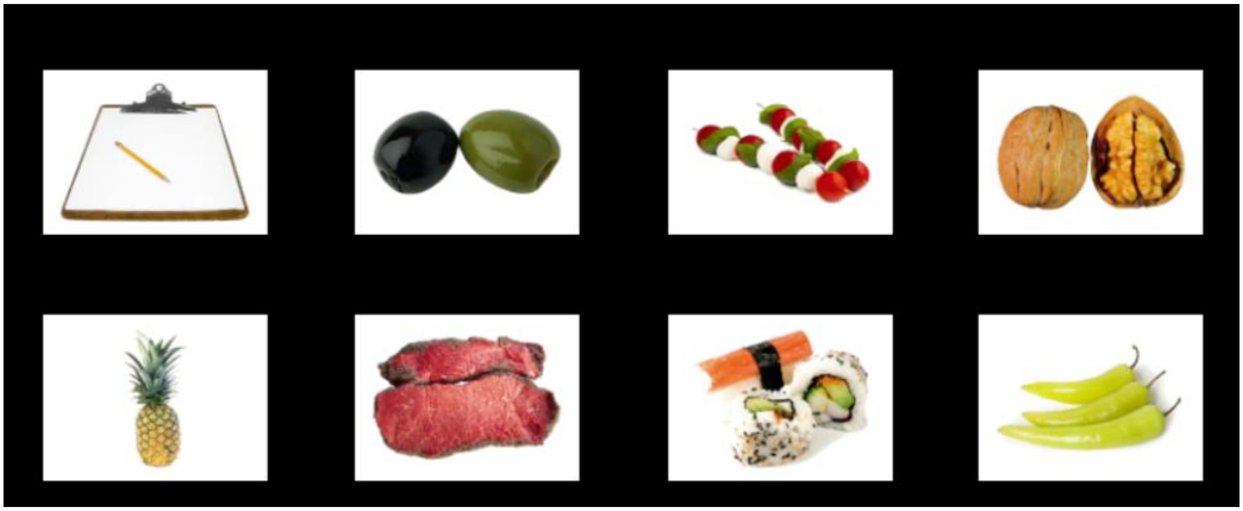

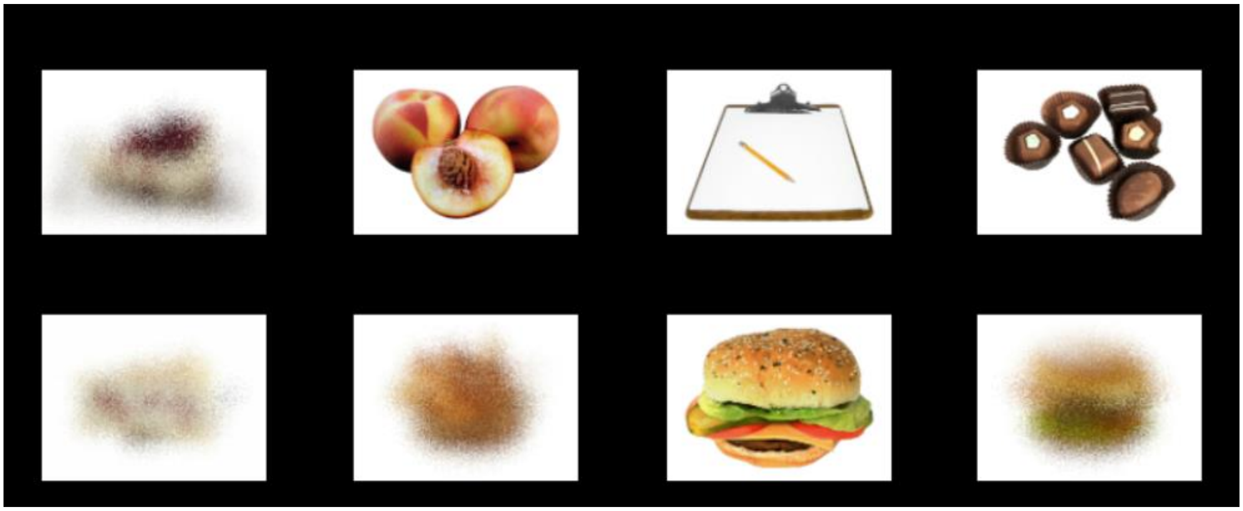

Supplement: Supplementary file 4 [file Data_Sheet_3.PDF]
